# Granzyme B contributes to subretinal fibrosis in neovascular age-related macular degeneration by modulating inflammation and epithelial-mesenchymal transition

**DOI:** 10.1186/s12974-025-03619-9

**Published:** 2025-11-27

**Authors:** Hyung-Suk Yoo, Harshini Chakravarthy, Jeanne Xi, Jing Cui, Zhengyuan Ai, Amir Hosseini, Jun Song, Neilan Tan, Natalie Ma, Ceres Zhou, Boaz Li, Robert Bell, Anne Haegert, Stephane Le Bihan, Myeong Jin Ju, David J. Granville, Joanne A. Matsubara

**Affiliations:** 1 Department of Ophthalmology and Visual Sciences, UBC, 2550 Willow St. Room 375 (North Side), Vancouver, BC V5Z 3N9 Canada; 2 School of Biomedical Engineering, UBC, Vancouver, BC Canada; 3 Vancouver Prostate Centre, Vancouver General Hospital, Vancouver, BC Canada; 4 M.H. Mohseni Institute of Urologic Sciences, Vancouver Coastal Health Research Institute, Vancouver, BC Canada; 5 International Collaboration on Repair Discoveries (ICORD), Vancouver Coastal Health Research Institute, Vancouver, BC Canada; 6 Department of Pathology and Laboratory Medicine, UBC, Vancouver, BC Canada

## Abstract

**Background:**

More than half of patients with neovascular age-related macular degeneration (nAMD) develop subretinal fibrosis, regardless of anti-vascular endothelial growth factor (VEGF) therapy. No treatment exists for subretinal fibrosis, as its pathophysiology remains elusive. Granzyme B (GzmB) is a serine protease elevated in human eyes with nAMD and contributes to choroidal neovascularization (CNV). Although GzmB is involved in dermal and cardiac fibrosis, its role in subretinal fibrosis has yet to be elucidated.

**Methods:**

Using the two-stage laser-induced mouse model of subretinal fibrosis, fibrotic lesions were induced in younger (3–6 months old) and older (7–14 months old) C57BL/6J and GzmB deficient mice. Seven days after the second laser, in vivo imaging of fibrotic lesions was performed using custom-built polarization diversity-optical coherence tomography (PD-OCT) system. Eyes were collected and used for either retina wholemounts or cross-sections. Wholemounts were immunostained to assess pathological features of fibrosis and determine the size of fibrotic lesions and mast cell counts within fibrotic lesions. Cross-sections were processed to quantify the levels of GzmB substrates within fibrotic lesions, namely pro-fibrotic thrombospondin-1 (TSP-1) and anti-fibrotic decorin (DCN), the extent of macrophage-to-myofibroblast transition (MMT), activation of astrocytes/Müller cells and finally photoreceptor cell death. ARPE-19 wound healing assay was performed to study the direct role of GzmB in RPE wound healing in vitro. GzmB-mediated transcriptional changes in ARPE-19 were determined by performing bulk RNA sequencing. Immunocytochemistry was performed to assess epithelial-mesenchymal transition (EMT).

**Results:**

GzmB deficiency resulted in smaller fibrotic lesions in older mice and was associated with decreased levels of TSP-1 and increased levels of DCN within fibrotic lesions. It also led to increased MMT but reduced mast cell counts within fibrotic lesions, which was correlated with reduced photoreceptor cell death. In ARPE-19, exogenous application of GzmB impaired wound closure and promoted partial EMT by selectively modulating genes involved in transforming growth factor-β signaling, EMT, inflammation, angiogenesis and cell-cell interaction.

**Conclusions:**

Our study reveals that extracellular GzmB is a key contributor to subretinal fibrosis in nAMD, modulating inflammation, EMT and photoreceptor degeneration. These findings suggest GzmB as a promising therapeutic target for mitigating the development of subretinal fibrosis in nAMD.

**Supplementary Information:**

The online version contains supplementary material available at 10.1186/s12974-025-03619-9.

## Introduction

Age-related macular degeneration (AMD) is the most common, blinding neurodegenerative eye disease that affects approximately 200 million people worldwide. It primarily affects individuals over the age of 55, and with the growing aging population, its prevalence is expected to rise significantly [1]. While considered a single disease, AMD can progress into either degenerative or proliferative forms. The degenerative form of AMD, known as “dry” AMD, accounts for 90% of AMD cases while the proliferative form, known as “wet” AMD or neovascular AMD (nAMD), affects only 10% of AMD patients [2]. Although nAMD represents a small proportion of total AMD cases, it accounts 90% of the blindness associated with AMD [2].

nAMD is caused by abnormal vascular growth in the choroid, known as choroidal neovascularization (CNV) [3]. The new vessels originating from the choroid breach the retinal pigment epithelium and Bruch’s membrane, both of which are essential component of the outer blood-retina barrier, and invade the subretinal space [4]. These vessels eventually leak exudates into the neuroretina and trigger photoreceptor degeneration, resulting in vision loss in patients with nAMD [2]. While the exact pathogenesis of nAMD is not fully understood, vascular endothelial growth factor (VEGF) has been identified as one of the main drivers of CNV, and intravitreal anti-VEGF therapy is the current gold-standard treatment for nAMD [5].

Emerging evidence suggests that more than half of patients with nAMD eventually develop a thick, permanent scar in the retina, known as subretinal fibrosis, despite of anti-VEGF treatments [4, 6]. This scar formation expands over time, exacerbates retinal neurodegeneration, and lowers visual acuity in patients with nAMD [4]. There is currently no treatment option for subretinal fibrosis, as its pathophysiology remains elusive [4]. However, it was recently proposed that there is a pathological transition from CNV to subretinal fibrosis, referred to as the angiofibrotic switch [7, 8]. Targeting molecules involved in this pathological transition may hold therapeutic value for preventing subretinal fibrosis in nAMD.

Here, we explore the role of a serine protease, Granzyme B (GzmB), a member of the Granzyme family often characterized by their cytotoxic roles in immune cell-mediated target cell death [9, 10]. GzmB is the most extensively studied member of this family. Beyond T cells and natural killer cells, GzmB expression can be induced in a variety of immune and non-immune cells, including choroidal mast cells and retinal pigment epithelium (RPE) cells in the outer retinal layers of the eye [9]. We have previously found not only that GzmB levels increase with age in the RPE and choroid of human, non-human primate and mouse eyes but also that GzmB is significantly elevated in the outer retinal layers of human eyes with CNV, compared to age-matched healthy human eyes [5, 9]. Furthermore, GzmB can degrade essential extracellular matrix (ECM) proteins and tight junctions that maintain the outer blood-retinal barrier and the integrity and function of the RPE and choroid [9]. Hypothesizing that GzmB disrupts the outer retinal layers and promotes CNV, we previously investigated the role of GzmB in CNV and found: (1) exogenous GzmB induces choroidal sprouting in RPE-choroid explants by cleaving anti-angiogenic proteins thrombospondin-1 (TSP-1) and decorin (DCN) and increasing VEGF release into the supernatant [3, 5] and (2) GzmB knockout (KO) in mice attenuates laser-induced CNV lesions in vivo [5].

GzmB also plays an important role in fibrosis. GzmB KO results in reduced collagen-1 (Col1) deposition in the heart [11], increased levels of anti-fibrotic protein DCN in the skin [12], and smaller fibrotic lesions in the skin and heart [11, 12]. Furthermore, topical application of a GzmB-specific inhibitor VTI-1002 prevents fibrotic scarring on the skin by preventing DCN cleavage and enhances DCN protein levels in the wound [13]. These findings together support the hypothesis that extracellular GzmB may contribute to subretinal fibrosis in nAMD. In this study, we utilized a two-stage laser-induced mouse model of subretinal fibrosis and found that GzmB deficiency results in significantly smaller subretinal fibrotic lesions in older mice. Immunofluorescence analyses revealed that: (1) GzmB deficiency leads to decreased TSP-1 and increased DCN immunolabeling within fibrotic lesions, (2) GzmB deficiency increases the overall accumulation of macrophages and MMT within fibrotic lesions but results in reduced accumulation of mast cells, and (3) GzmB deficiency reduces photoreceptor cell death in response to subretinal fibrosis. Finally, using ARPE-19, we demonstrated that exogenous GzmB impairs wound healing responses and promotes partial EMT, which was supported by bulk RNA sequencing and immunocytochemistry.

## Results

### GzmB deficiency reduces the size of subretinal fibrotic lesions in older mice

**Fig. 1:**
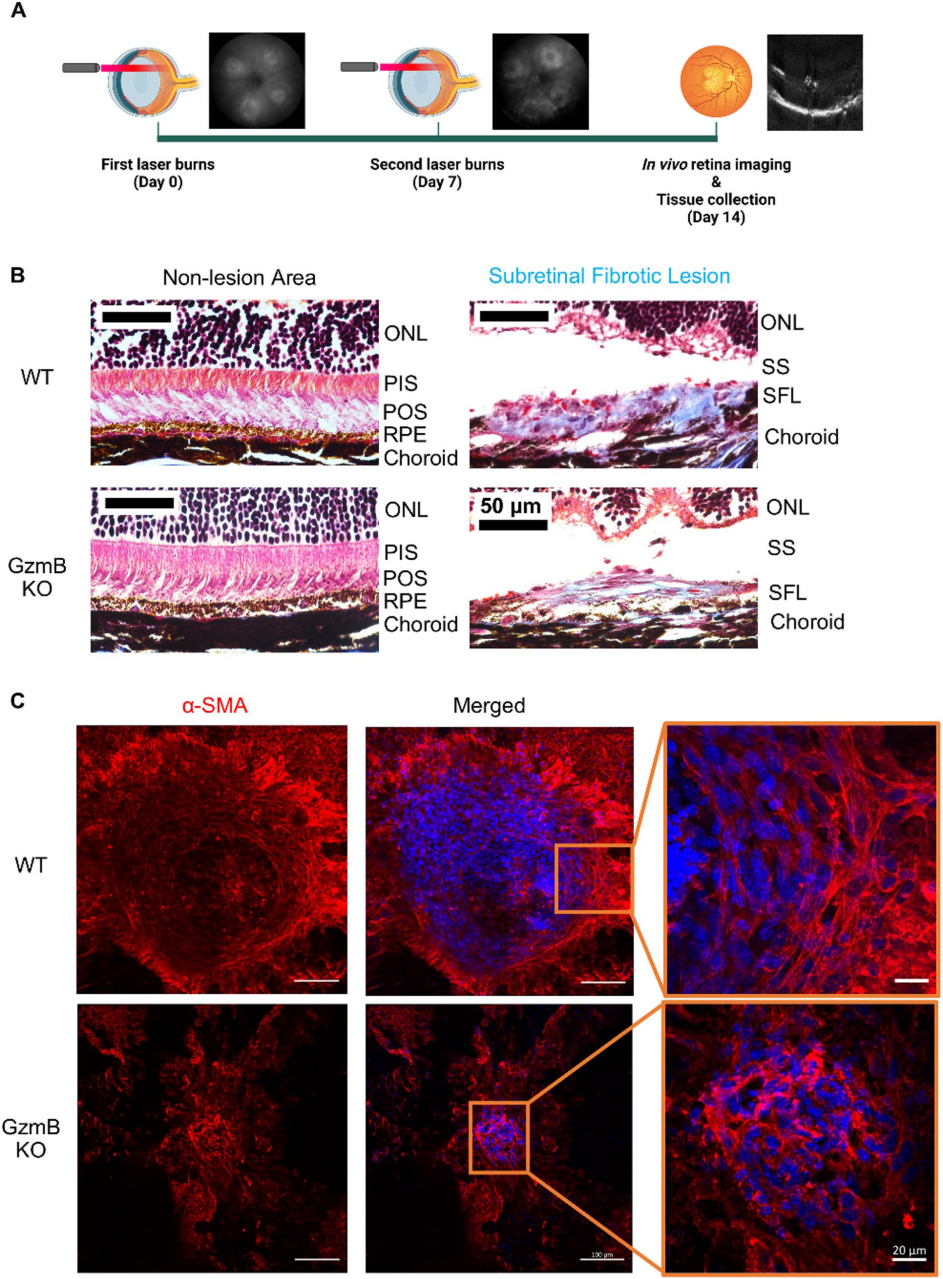
Two-stage laser-induced mouse model of subretinal fibrosis results in pathological features of fibrosis in the eye. **A** Experimental timeline of two-stage laser-induced subretinal fibrosis in mice. Fundus images show how laser sites look one weak after each laser burn. **B** Masson’s trichrome staining on non-lesion sites and subretinal fibrotic lesions in WT and GzmB KO mice. Collagen deposition within subretinal fibrotic lesions appears blue. Scale bars, 50 μm. **C** Subretinal fibrotic lesions in RPE-choroid wholemounts from WT and GzmB KO mice showing that the fibrotic lesions contain cells (DAPI; blue) expressing α-SMA (red). The boxed areas in subretinal fibrotic lesions are shown in higher magnification to the right. Scale bars, 100 μm and 20 μm. *ONL* Outer Nuclear Layer, *PIS* Photoreceptor Inner Segment, *POS* Photoreceptor Outer Segment, *RPE* Retinal Pigment Epithelium, *SS* Subretinal Space, *SFL* Subretinal Fibrotic Lesion

Subretinal fibrotic lesions were induced in WT and GzmB KO mice using the two-stage laser-induced mouse model of subretinal fibrosis [14], implemented with the Micron IV laser system (250 mW power, 100 ms duration, 50 μm spot size), which we previously used to induce CNV lesions [15] (Fig. 1A). Masson’s trichrome staining confirmed collagen accumulation within subretinal fibrotic lesions (Fig. 1B). α-SMA+ cells were present in lesions from both WT and GzmB KO mice (Fig. 1C). These cells exhibited spindle-shaped morphology and bundled α-SMA+ fibers characteristic of myofibroblasts [16]. Some α-SMA+ cells also co-expressed fibronectin, a well-known marker for EMT and fibrosis [4, 17] (Fig. S1A). CD31, a well-established marker of endothelial cells and angiogenesis [15], was minimally detected within the fibrotic lesions induced by the Micron IV laser system, suggesting limited endothelial content and reduced angiogenic activity in this model of subretinal fibrosis (Fig. S1B).

**Fig. 2:**
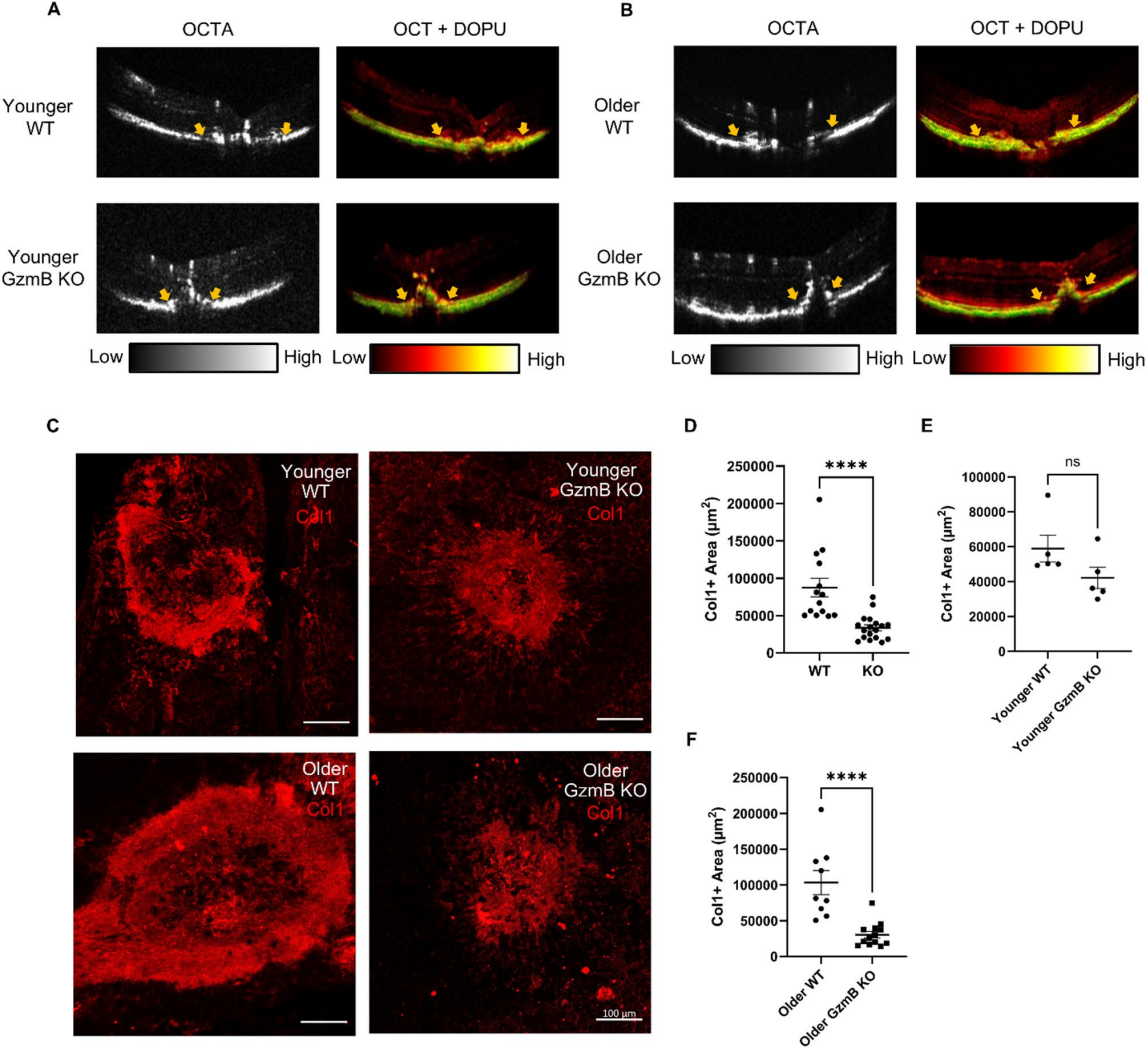
GzmB deficiency attenuates subretinal fibrosis in older mice. **A**, **B** Representative PD-OCT images of subretinal fibrotic lesions in younger (3–6 month) (**A**) and older (7–14 month) (**B**) WT and GzmB KO mice. **C** Representative confocal images of collagen-1+ subretinal fibrotic lesions (red) in younger and older WT and GzmB KO mice. **D**, **E**, **F** Quantitative measurements of collagen-1+ lesion areas in younger (*n* = 5 animals per group) and older (*n* = 9–13 per group) WT and GzmB KO mice. Scale bar, 100 μm. The data are presented as mean ± SEM. Statistical analyses were performed by Mann-Whiteny U test, ****p < 0.0001

Cross-sectional optical coherence tomography (OCTA) images revealed laser-induced angiogenic responses and neovascularization in the choroid across fibrotic lesions. Additionally, the composite OCT and degree of polarization uniformity (DOPU) images reveal the morphological disruptions caused by laser induction. Depolarization contrast further emphasizes these disruptions, which selectively highlights melanin molecules in the RPE and choroid [15, 18]. WT mice exhibited wider deformations in the traversal direction, whereas GzmB KO mice showed elevated lesions in the axial direction with smaller traversal deformations (Fig. 2A, B).

Subretinal fibrotic lesion area was quantified using Col1 immunostaining. Across all ages, the mean area of Col1+ lesions in GzmB KO mice (33675 ± 3906 µm^2^) was significantly smaller than in WT mice (87391 ± 12331 µm^2^) (Fig. 2C, D). Mice were categorized into younger (3–6 months) and older (7–14 months) groups. In older mice, the mean lesion area in GzmB KO mice (30331 ± 4676 µm^2^) was significantly smaller than in WT mice (103184 ± 16748 µm^2^) (Fig. 2C, F). In younger mice, the mean lesion area in GzmB KO mice (42161 ± 6158 µm^2^) was smaller than in WT mice (58962 ± 7727 µm^2^), but the difference was not statistically significant (Fig. 2C, D).

In WT mice, older animals exhibited significantly larger lesion areas than younger animals (Fig. S2A). In GzmB KO mice, lesion areas did not differ significantly between age groups (Fig. S2B). Among WT mice, lesion area differed significantly between males and females (Fig. S2C), whereas no sex-based differences were observed in GzmB KO mice (Fig. S2D). Because we only observed a significant size difference in Col1+ fibrotic lesions between older WT and GzmB KO mice, we decided to use older WT and GzmB KO mice for the rest of the study. Hereafter, WT and GzmB KO mice refer to mice in the older age group.

### GzmB deficiency results in reduced TSP-1 and elevated DCN immunolabeling within subretinal fibrotic lesions

TSP-1 and DCN were selected for analysis based on prior evidence identifying them as proteolytic substrates of GzmB [3, 15]. GzmB-mediated cleavage of TSP-1 reduces its anti-angiogneic activity while promoting choroidal sprouting [3, 15]. However, the TSP-1 fragments, including type-1 repeats and the N-terminal domain, have been implicated in fibrosis through mechanisms such as latent TGF-β activation and collagen deposition [19]. Of note, the N-terminal domain fragment can be generated by GzmB [3]. DCN exhibits both anti-angiogenic and anti-fibrotic properties, and previous studies have shown that genetic deletion or pharmacological inhibition of GzmB can prevent DCN cleavage and increase DCN levels in several skin disorders [12, 13]. To evaluate the distribution of these ECM proteins within subretinal fibrotic lesions, we investigated the immunoreactivity of TSP-1 and DCN in WT and GzmB KO mice.

**Fig. 3:**
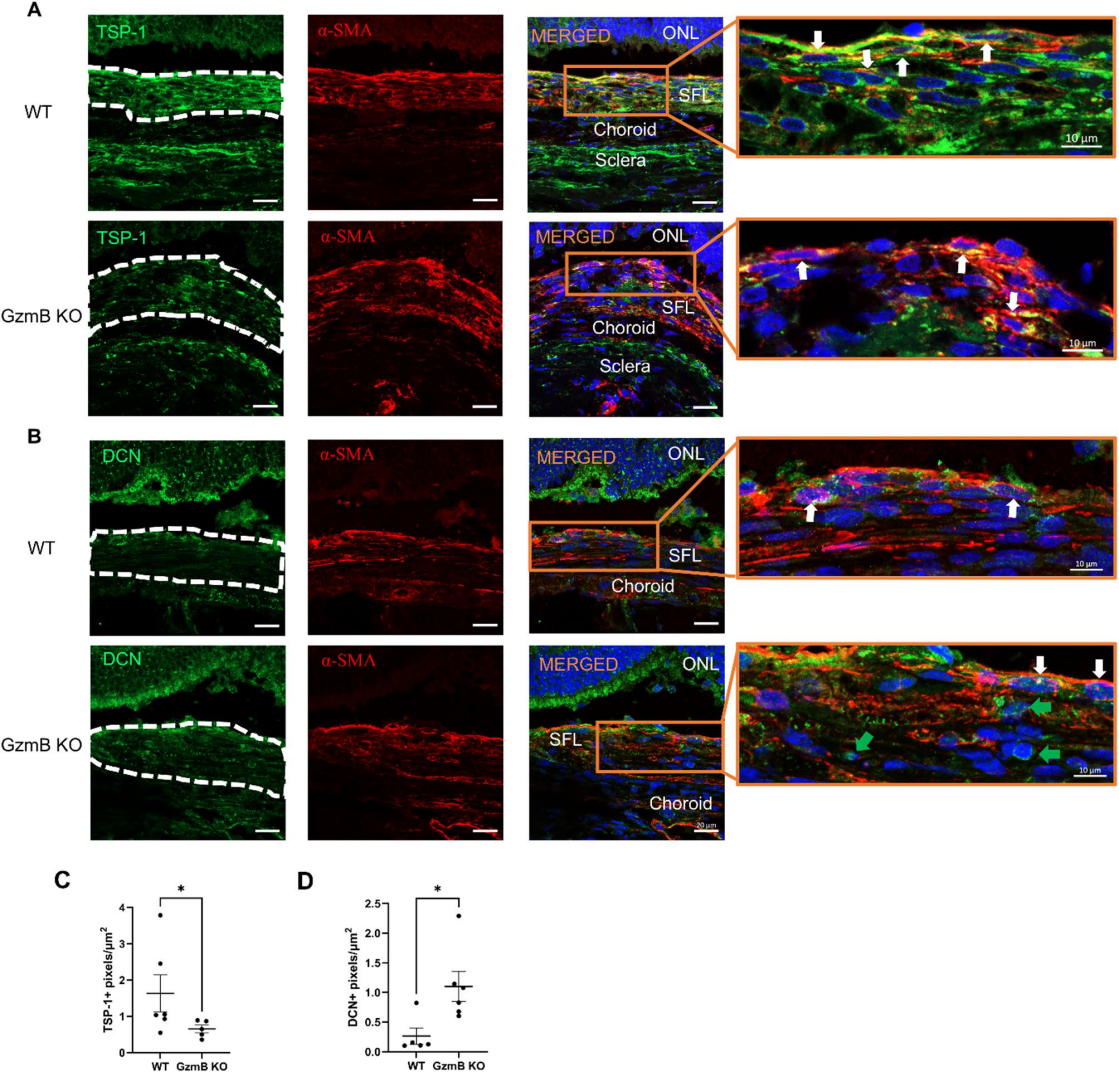
GzmB KO decreases TSP-1 and increases DCN immunolabeling within subretinal fibrotic lesions. **A**, **B** Representative confocal images of non-lesion sites and subretinal fibrotic lesions in retinal cross-sections from older WT and GzmB KO mice (*n* = 5–6 animals per group) showing TSP-1 (**A**) or DCN (**B**) (green), α-SMA (red) and cell nuclei (blue). Dashed lines indicate the boundaries of α-SMA + subretinal fibrotic lesions. Blow-up images show cells co-expressing TSP-1/DCN (green) and α-SMA (red) (white arrows) and cells only expressing DCN (green arrows). Scale bars, 20 μm and 10 μm. **C**, **D** Pixel count analyses of TSP-1 (**C**) and DCN (**D**) immunolabeling within subretinal fibrotic lesions. The data are presented as mean ± SEM. Statistical analyses were performed by Mann-Whiteny U test, *p < 0.05. *ONL* Outer Nuclear Layer, *SFL* Subretinal Fibrotic Lesion

Subretinal fibrotic lesions in WT mice exhibited higher TSP-1 immunolabeling compared to those in GzmB KO mice (Fig. 3A, C). Most α-SMA+ cells within the fibrotic lesions expressed TSP-1 in both genotypes (Fig. 3A). In naïve eyes, TSP-1 immunolabeling was weakly detected in the inner and outer nuclear layers, photoreceptor inner segment, and RPE, with comparatively stronger labeling in the photoreceptor outer segment. α-SMA immunolabeling was not present in the outer retina (Fig. S3B).

On the contrary, fibrotic lesions in GzmB KO mice exhibited increased DCN immunolabeling compared to WT mice (Fig. 3B, D). α-SMA+ cells expressing DCN were present in both genotypes; however, additional DCN+ cells lacking α-SMA immunolabeling were observed within fibrotic lesions of GzmB KO mice (Fig. 3B). In naïve eyes, weak DCN immunolabeling was detected in the inner and outer nuclear layers and photoreceptor inner segment of both WT and GzmB KO mice, with comparatively stronger labeling in the photoreceptor outer segment. α-SMA immunolabeling was not present in the outer retina (Fig. S3B). Negative control immunolabeling confirmed antibody specificity (Fig. S3A).

### GzmB deficiency increases IBA1 + macrophage accumulation and promotes macrophage-to-mesenchymal transition in subretinal fibrotic lesions

**Fig. 4:**
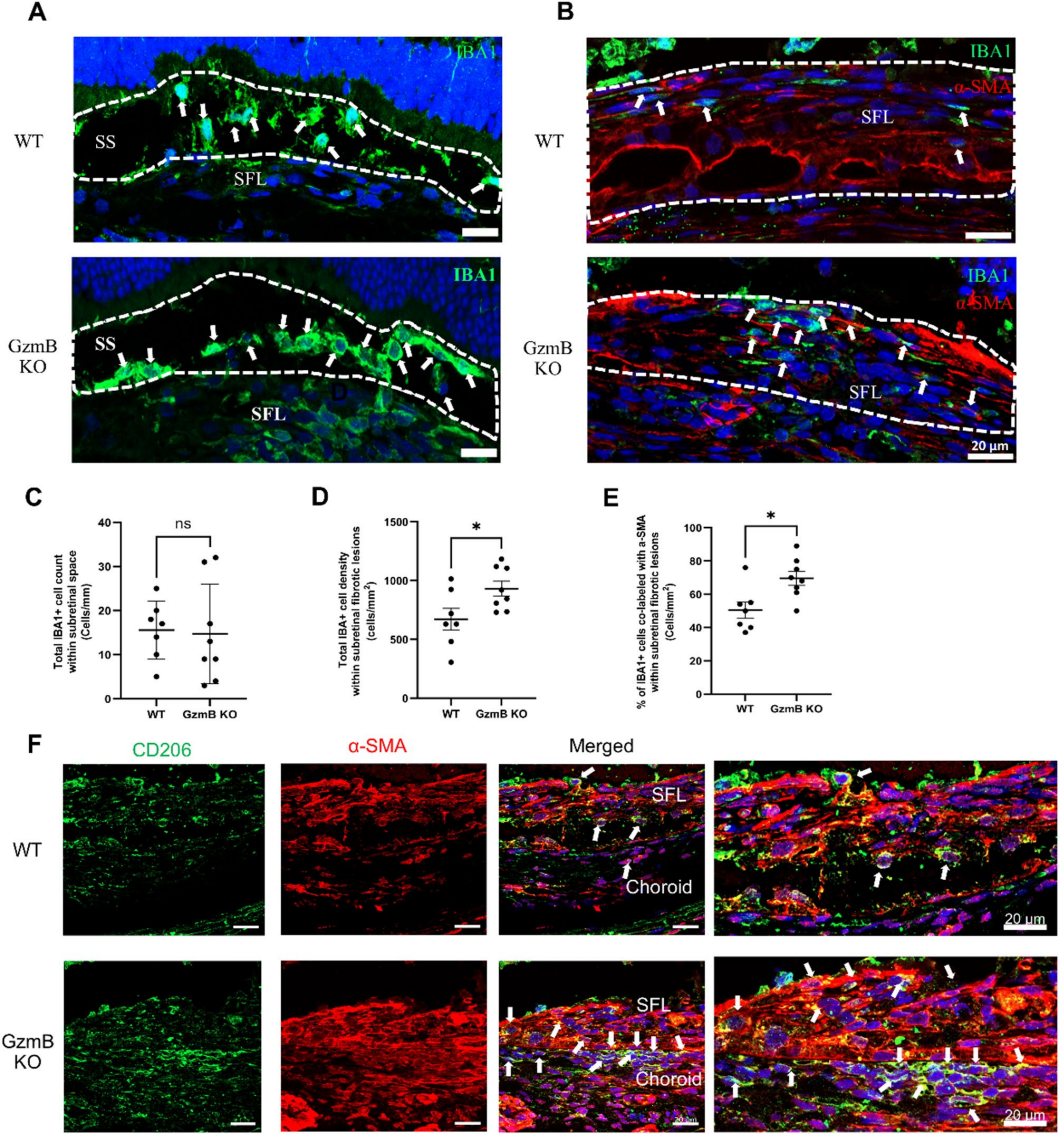
GzmB KO prevents neither accumulation of IBA1 + cells in the subretinal space above subretinal fibrotic lesions and within subretinal fibrotic lesions nor macrophage-mesenchymal transition within subretinal fibrotic lesions but promotes the M1-to-M2 transition. **A**,** C** Immunolabeling (**A**) and counting (**C)** of IBA1+ cells (white arrows) in the subretinal space above subretinal fibrotic lesions in older WT and GzmB KO mice (*n* = 7-8 animals per group). **B**, **D**, **E** Immunolabeling (**B**) and counting (**D**, **E**) of total IBA1+ cells and IBA1+ α-SMA+ cells (white arrows) within subretinal fibrotic lesions in older WT and GzmB KO mice (*n* = 7-8 animals per group). (**F**) Immunolabeling of CD206+ α-SMA+ cells (white arrows) within and below subretinal fibrotic lesions in older WT and GzmB KO mice. Blow-up images of CD206+ α-SMA+ cells (white arrows) are shown on the right. Scale bars, 20 μm. The data are presented as mean ± SEM. Statistical analyses were performed by Mann-Whiteny U test. *SS* Subretinal Space, *SFL* Subretinal Fibrotic Lesion

Given the strong immunolabeling of TSP-1, a putative chemoattractant for macrophages and mast cells [20-23], within subretinal fibrotic lesions in WT mice, we assessed whether GzmB deficiency alters the accumulation of these immune cells. Macrophages were identified using IBA1 immunolabeling and found to accumulate both within subretinal fibrotic lesions and in the subretinal space above the lesions (Fig. 4). The total IBA1+ cell count in the subretinal space did not differ significantly between WT and GzmB KO mice (Fig. 4A, C). However, IBA1+ cell density within fibrotic lesions was significantly higher in GzmB KO mice compared to WT mice (Fig. 4B, D). A higher proportion of IBA1+ cells expressed α-SMA within fibrotic lesions in GzmB KO mice (Fig. 4B, E).

To further characterize macrophage phenotype, CD206 immunolabeling was used to identify M2 macrophages, which are known to promote tissue repair and can transdifferentiate into myofibroblasts via macrophage-to-myofibroblast transition (MMT) [24, 25]. CD206+ α-SMA+ cells were present within fibrotic lesions in both WT and GzmB KO mice. However, GzmB KO mice exhibited a greater number of CD206+ α-SMA+ cells within fibrotic lesions and in the choroid directly beneath the lesions (Fig. 4F).

### GzmB deficiency reduces the accumulation of mast cells within subretinal fibrotic lesions and reduces photoreceptor cell death in response to subretinal fibrosis

**Fig. 5:**
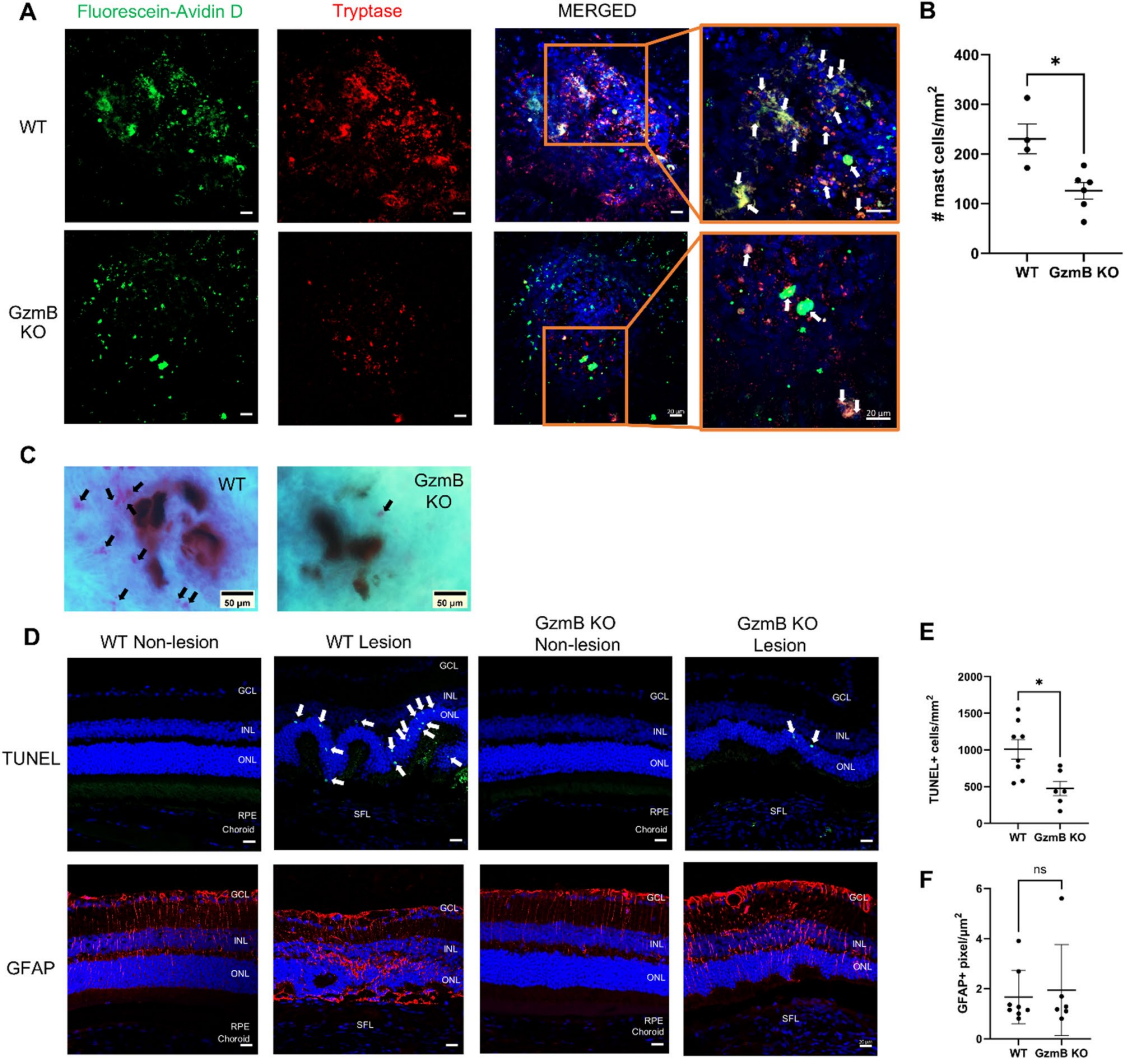
GzmB KO reduces the accumulation of mast cells within subretinal fibrotic lesions and increases photoreceptor survival and reactivity of macroglia. A Representative confocal images of similarly sized subretinal fibrotic lesions in RPE-choroid wholemounts from older WT and GzmB KO mice showing fluoresceine-avidin D-labeled mast cells (green), tryptase (red) and cell nuclei (blue). The boxed areas in subretinal fibrotic lesions are shown in higher magnification to the right. Fluoresceine-avidin D-labeled and tryptase+ cells are indicated by white arrows. Scale bars, 20 µm. B Mast cell count within each subretinal fibrotic lesion in older WT and GzmB KO mice (n = 4-6 animals per group). C Toluidine blue staining of subretinal fibrotic lesions in cleared RPE-choroid wholemounts from older WT and GzmB KO mice. Mast cells appear pink/purple (black arrows) in subretinal fibrotic lesions. Scale bar, 50 µm. D, E, F Representative confocal images (D) and quantification (E, F) of the retina cross-sections showing TUNEL (green) and GFAP (red) staining in the neuroretina directly above subretinal fibrotic lesions in WT and GzmB KO mice (n = 6-8 animals per group). TUNEL+ cells in ONL are indicated by white arrows. The data are presented as mean ± SEM. Statistical analyses were performed by Mann-Whiteny U test, *p < 0.05. GCL = Ganglion Cell Layer, INL = Inner Nuclear Layer, ONL = Outer Nuclear Layer, RPE = Retinal Pigment Epithelium, SFL = Subretinal Fibrotic Lesion

Mast cells were assessed within subretinal fibrotic lesions based on prior evidence identifying them as a significant source of GzmB in the outer retina [9] and their known accumulation in fibrotic lesions of other organs [26–28]. To label mast cells, we used fluorescein-conjugated avidin D, which binds specifically to mast cell granules [29], and co-immunostained for tryptase, a mast cell-specific protease implicated in fibrosis [30, 31]. Both WT and GzmB KO mice exhibited fluorescein-avidin D-labeled and tryptase+ mast cells within subretinal fibrotic lesions (Fig. 5A). Tryptase co-localized with fluorescein-avidin D and DAPI, but it was primarily observed as degranulated products. Fluorescein-avidin D showed stronger co-localization with DAPI and was used as the primary marker for mast cell quantification. GzmB KO mice exhibited fewer mast cells within subretinal fibrotic lesions compared to WT mice (Fig. 5B).

Toluidine blue staining of RPE-choroid wholemounts was used to further confirm mast cell presence. Mast cells appeared as purple/pink cells, while fibrotic lesions were identified by round black tissue debris created by laser energy. Consistent with immunofluorescent data, toluidine blue staining revealed fewer mast cells in subretinal fibrotic lesions of GzmB KO mice compared to WT mice (Fig. 5C).

To assess photoreceptor cell survival and macroglial activation in the neuroretina above subretinal fibrotic lesions, we performed TUNEL and GFAP staining. TUNEL staining revealed significantly fewer dead or dying photoreceptor cells in the ONL above fibrotic lesions in GzmB KO mice (Fig. 5D). GFAP immunoreactivity was not significantly different between WT and GzmB KO mice (Fig. 5E). GFAP immunoreactivity was also present in non-lesion sites nearby the fibrotic lesions, with labeling observed throughout the neuroretina (Fig. 5D). This pattern stands in stark contrast to the weak GFAP immunolabeling limited to the ganglion cell layer (GCL) observed in naïve eyes (Fig. S4A). GFAP+ macroglia were also present above subretinal fibrotic lesions in both genotypes (Fig. S4B).

### Exogenous GzmB impairs wound closure in ARPE-19, and the GzmB-specific inhibitor VTI-1002 rescues wound closure

To investigate how GzmB may directly contribute to subretinal fibrosis, we utilized an ARPE-19 wound healing assay. RPE cells were selected based on prior evidence that GzmB can cleave tight junction proteins (ZO-1), cell adhesion molecules (JAM-A, occludin), and ECM components (laminin, fibronectin, collagen IV) expressed by ARPE-19 cells [9]. TGF-β2, the predominant isoform associated with retinal fibrosis [4], was used as a positive control for EMT and wound closure, given its well-established role in these two processes [32].

**Fig. 6:**
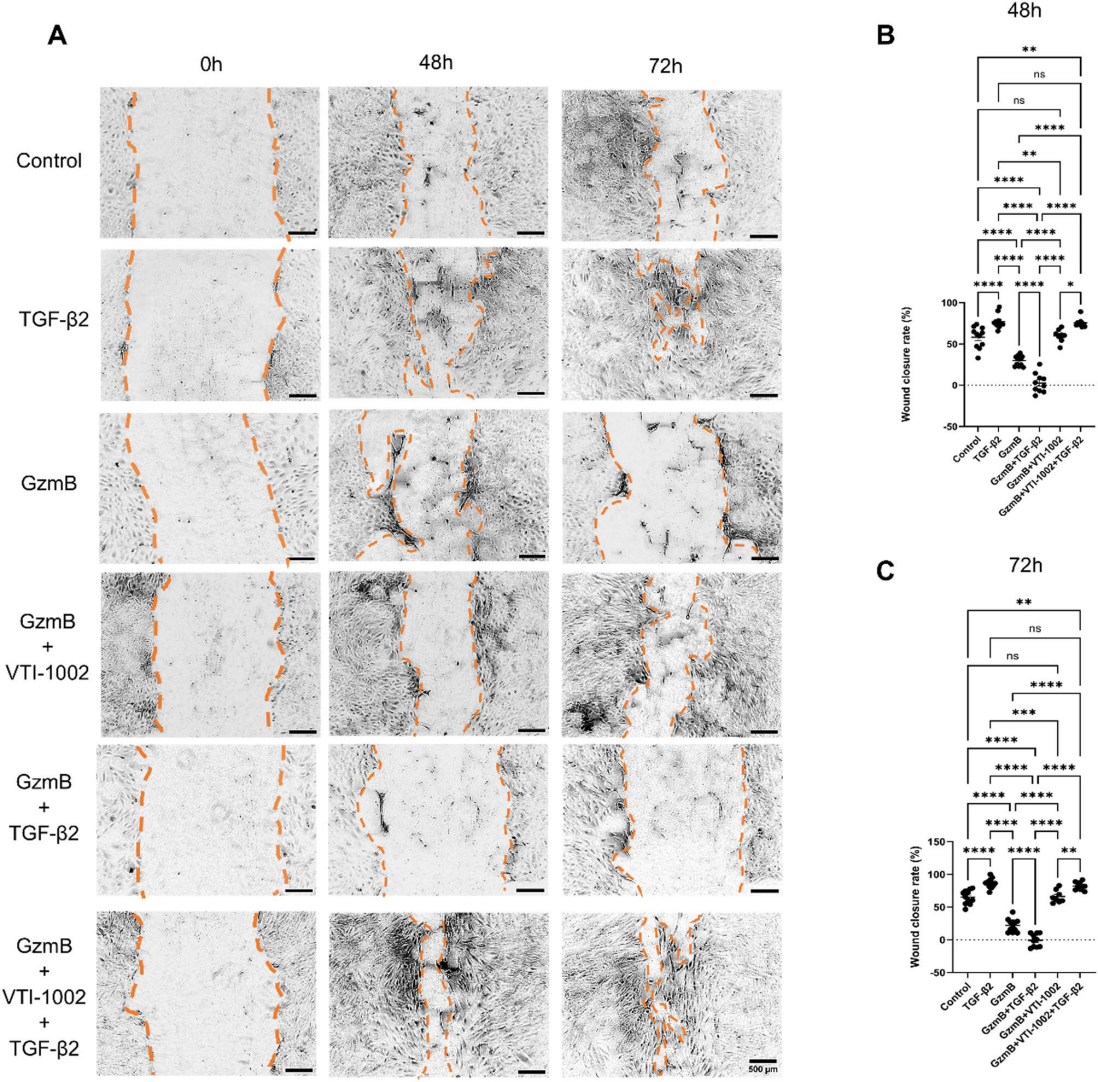
GzmB delays wound closure in ARPE-19. **A** Representative phase-contrast images of scratch wounds in each treatment group at 0-, 48- and 72-hour timepoints. The borders of wound edges are indicated by orange dotted lines. Scale bar, 500 μm. **B**, **C** ARPE-19 wound closure rate in each treatment group at 48 and 72 h (*n* = 8–12 samples per group; representative of three-independent experiments). The data are presented as mean ± SEM. Statistical analyses were performed by one-way ANOVA Tukey’s multiple comparison test, *p < 0.05, **p < 0.01, ***p < 0.001 and ****p < 0.0001

GzmB treatment significantly delayed wound closure compared to control and TGF-β2 treatments at 24, 48, and 72 h (Fig. 6A-C; Fig. S5A, B). Co-administration of GzmB and TGF-β2 further delayed wound closure relative to control and TGF-β2 alone at all timepoints, and compared to GzmB alone at 48 and 72 h. The co-treatment also resulted in mixed patterns of wound expansion and closure at 48 and 72 h (Fig. 6A-C). To investigate whether GzmB affects cell migration and proliferation, we assessed β-catenin localization in ARPE-19 cells at the wound edges, as it accumulates in cytoplasm and eventually enters the nucleus during both cell migration and proliferation [33]. In control and TGF-β2-treated groups, cells at the wound edge exhibited elongated morphology with cytoplasmic and nuclear β-catenin immunolabeling, α-SMA expression, and reduced or internalized ZO-1 (Fig. S6A, B). These groups also showed a greater number of cells that appeared detached from the wound edge. In contrast, GzmB-stimulated cells displayed elongated morphology and long α-SMA+ fibers at the wound edge, but showed mixed β-catenin localization, including membrane-associated, cytoplasmic and nuclear immunolabeling (Fig. S6A). ZO-1 immunolabeling was absent in cells at the immediate wound edge following GzmB treatment but was retained in some cells adjacent to the wound edge (Fig. S6B). In non-wounded ARPE-19, ZO-1 and β-catenin co-localized at the membrane with low immunolabeling of α-SMA, consistent with intact junctional architecture (Fig. S6A, B).

To assess whether GzmB inhibition could restore wound healing, we used VTI-1002, a small-molecule inhibitor of GzmB previously shown to accelerate wound closure in skin [13] and prevent choroidal sprouting in outer retina explants [15]. A low concentration of VTI-1002 (25 µM) significantly enhanced wound closure when administered with GzmB or GzmB + TGF-β2 at 48 and 72 h (Fig. 6A-C).

**Fig. 7:**
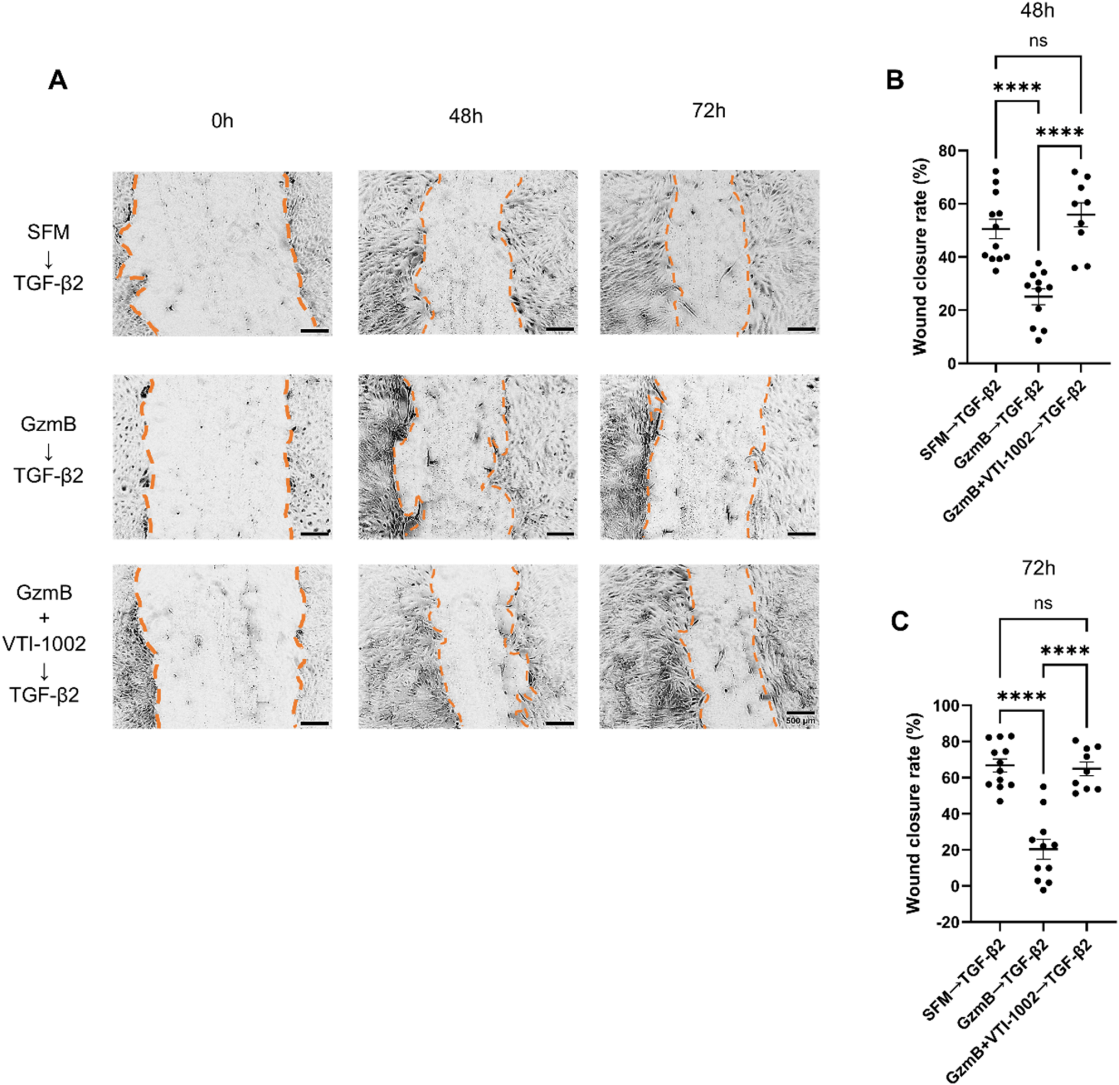
Acute GzmB stimulation delays TGF-β2-mediated wound closure in ARPE-19. **A** Representative phase-contrast images of scratch wounds in each treatment group at 0-, 48- and 72-hour timepoints. The borders of wound edges are indicated by orange dotted lines. **B**, **C** ARPE-19 wound closure rate in each treatment group at 48 (**B**) and 72 (**C**) hours (*n* = 9–12 samples per group; representative of three-independent experiments). The data are presented as mean ± SEM. Statistical analyses were performed by one-way ANOVA Tukey’s multiple comparison test, ****p < 0.0001

To determine if acute GzmB exposure could impair subsequent wound healing, ARPE-19 was stimulated with GzmB for 6 h followed by TGF-β2 for 24, 48, and 72 h. The GzmB → TGF-β2 group showed significantly delayed wound closure compared to serum-free medium (SFM) → TGF-β2 group at all timepoints (Fig. 7; Fig S6). Addition of VTI-1002 to the GzmB → TGF-β2 group significantly improved wound closure at 48 and 72 h (Fig. 7A-C).

### Acute GzmB stimulation alters EMT-related gene expression and cell phenotypes in ARPE-19 cells

**Fig. 8:**
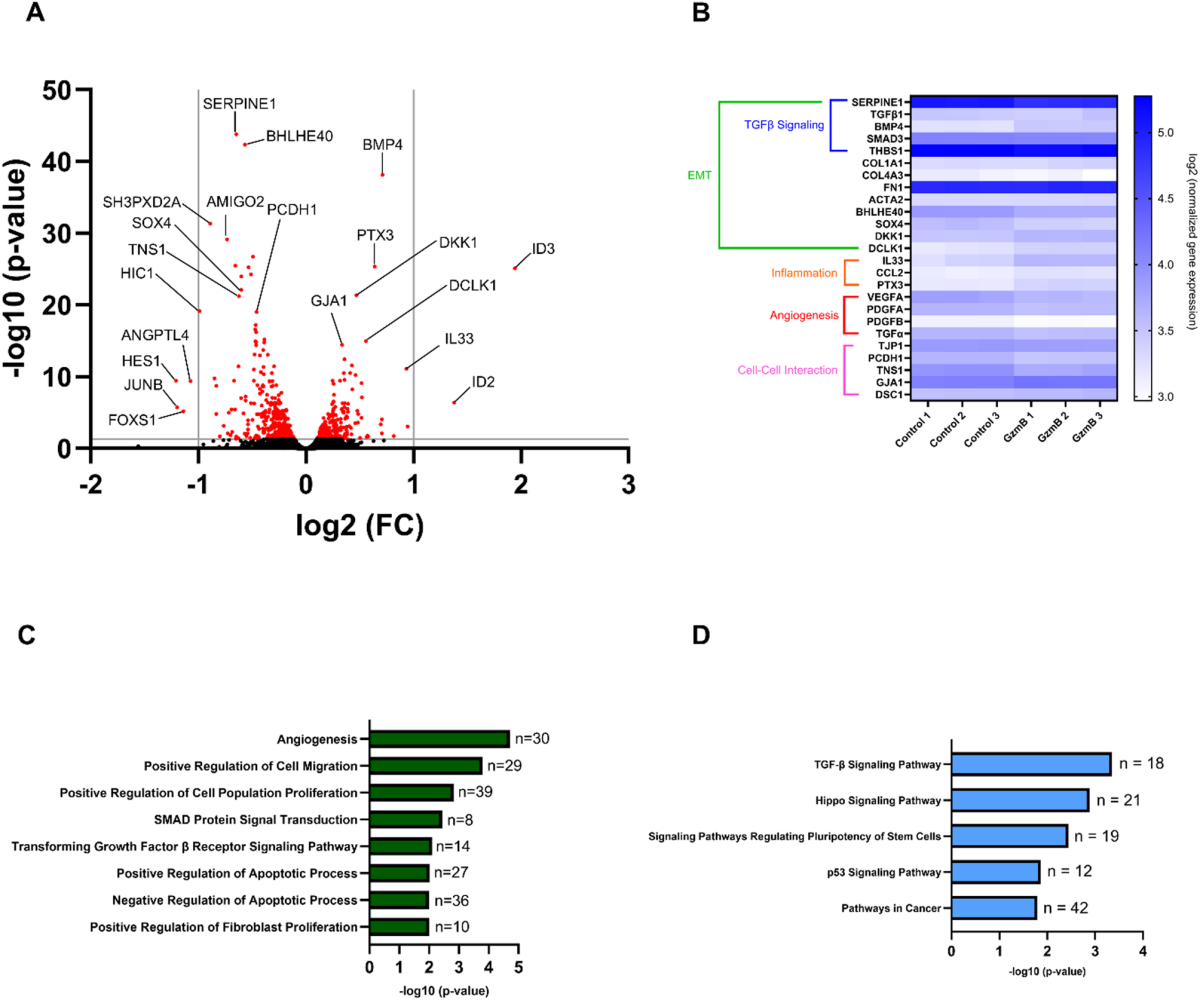
Acute GzmB stimulation alters gene expression profiles associated with TGF-β signaling, EMT, inflammation, angiogenesis and cell-cell interaction. **A** Volcano plot showing differentially expressed genes in ARPE-19 cells following acute GzmB stimulation (6 h, 100 nM) compared to controls (*n* = 3 per group). A total of 12,186 genes were analyzed, with significantly upregulated and downregulated genes highlighted in red. Selected genes involved in TGF-β signaling, EMT, inflammation, angiogenesis, and cell-cell interaction are labeled. Grey vertical lines indicate log_2_ (fold change) thresholds of ± 1.0, and the horizontal line indicates an adjusted p-value cutoff of 0.05. **B** Heatmap showing the relative expression of 25 selected genes associated with biological processes influenced by acute GzmB stimulation in ARPE-19 cells. Expression values are log2-transformed normalized counts. Genes were selected based on their involvement in biological processes influenced by GzmB, including TGF-β signaling, EMT, inflammation, angiogenesis, and cell-cell interaction. **C** Bar graph depicting significantly enriched Gene Ontology (GO) biological processes identified through DAVID functional annotation of 714 significant differentially expressed genes in ARPE-19 cells following acute GzmB stimulation. n = number of differentially expressed genes. **D** Bar graph showing significantly enriched KEGG pathways through DAVID functional annotation of 713 significant differentially expressed genes in ARPE-19 cells following acute GzmB stimulation. n = number of differentially expressed genes

Given that acute GzmB stimulation significantly delayed wound closure in ARPE-19, we investigated whether the effect was accompanied by transcriptional changes that could influence cellular behavior even in the presence of TGF-β2. Bulk RNA sequencing was performed on ARPE-19 following a 6-hour GzmB stimulation. Differential expression analysis revealed 713 significantly regulated genes. Volcano plot analysis showed downregulation of genes associated with TGF-β signaling (e.g. SERPINE1, BHLHE40, HES-1, JUNB, FOXS1, HIC1, SH3PXD2A, AMIGO2), angiogenesis (e.g. VEGFA, ANGPTL4), and cell-cell junctions (e.g. TNS1, PCDH1) (Fig. 8A). Upregulated genes included those involved in cell-cell junctions (e.g. GJA1, DSC1), EMT-related processes (e.g. BMP4, SOX4, ID2, ID3, DKK1, DCLK1), and inflammation (e.g. PTX3, IL33) (Fig. 8A).

A heatmap of 25 selected genes highlighted differential expression patterns between control and GzmB-treated samples, encompassing pathways related to TGF-β signaling, EMT, inflammation, and cell-cell interaction (Fig. 8B). To validate transcriptional changes identified by RNA sequencing, we performed quantitative PCR (qPCR) on a subset of genes associated with EMT, inflammation, and angiogenesis. qPCR analysis was conducted at both 6-hour and 18-hour timepoints following GzmB stimulation to assess temporal dynamics. BMP4, IL-33 and VEGF-A gene expression showed a significant increase only at 18 h, suggesting delayed accumulation of mRNA and potentially reflecting late-phase transcriptional activation (Fig. S8A, B, D). GJA1 gene expression was significantly elevated at both time points while SOX4 expression remained unchanged (Fig S8C, D). These findings largely reflect the transcriptional trends identified by RNA sequencing and reveal time-dependent regulation, underscoring the temporal complexity of GzmB-mediated gene expression. DAVID Gene Ontology (GO) enrichment analysis of the 713 differentially expressed genes revealed regulation of biological processes including angiogenesis, cell migration, cell proliferation, TGF-β signaling, and apoptosis (Fig. 8C). KEGG pathway analysis further supported these findings, identifying enrichment in TGF-β and EMT-related signaling pathways (Fig. 8D).

**Fig. 9:**
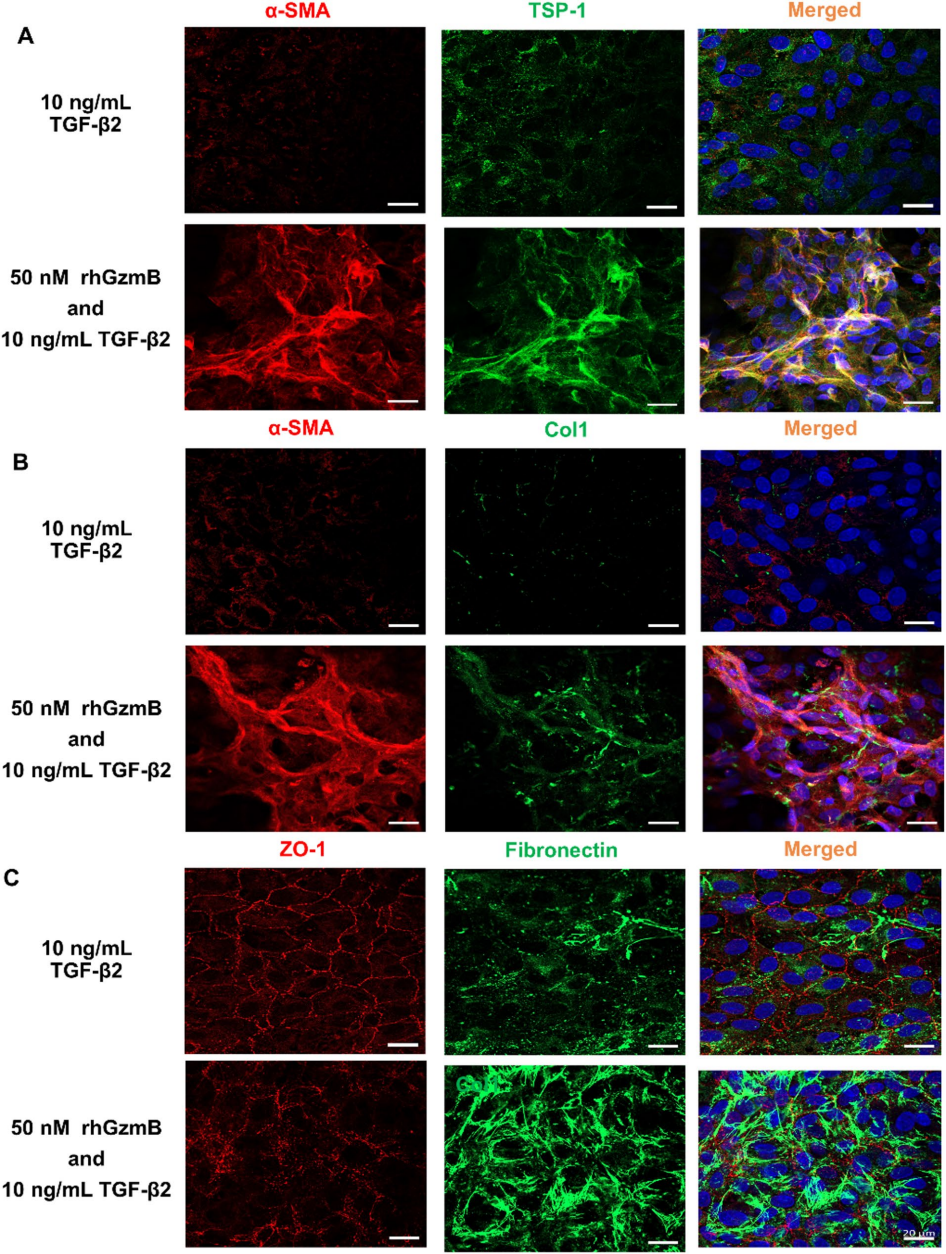
Acute GzmB stimulation leads to loss of ZO-1 and expression of EMT markers in ARPE-19 cells in the presence of TGF-β2. **A**, **B**, **C** Representative confocal images showing cellular morphology and immunostaining for α-SMA (red) (**A**, **B**), TSP-1 (green) (**A**), Col1 (green) (**B**), ZO-1 (red) (**C**), fibronectin (green) (**C**) and cell nuclei (blue) in ARPE-19 cells after 48-hour TGF-β2 treatment or after 6-hour 50 nM GzmB stimulation followed by 48-hour TGF-β2 treatment. Scale bar, 20 μm

Based on the mixed transcriptional profile, we hypothesized that GzmB may promote partial EMT and prevent full transdifferentiation of ARPE-19 cells into myofibroblasts in the presence of TGF-β2. Following a 6-hour GzmB stimulation and overnight incubation in 2% fetal bovine serum (FBS), clusters of cells co-expressing α-SMA and TSP-1 were observed on top of the ARPE-19 monolayer (Fig. S7B). These α-SMA+ TSP-1+ cells lacked typical myofibroblast morphology, such as spindle-shaped bodies and α-SMA+ stress fibers (Fig. S7C). When ARPE-19 cells were treated with TGF-β2 for 48 h following acute GzmB stimulation, clusters of cells exhibited loss of ZO-1 and expressed EMT markers including α-SMA, TSP-1, Col1, and fibronectin (Fig. 9A-C). Morphologically, these cells appeared more elongated and fibrous compared to cells stimulated with GzmB alone (Fig. S7C).

## Discussion

Subretinal fibrosis is a pathological phenomenon that develops secondary to CNV as part of the natural wound healing process [4]. However, chronic inflammation can disrupt this wound healing process, leading to excessive accumulation of ECM proteins secreted by myofibroblasts, such as Col1 and fibronectin [4]. Current anti-VEGF therapies for nAMD may suppress CNV, but do not prevent the pathological transition from CNV to subretinal fibrosis, which occurs in more than half of patients with nAMD [4, 6]. Hence, identifying molecules involved in the angiofibrotic switch could aid in developing therapeutic strategies against subretinal fibrosis. In our previous studies, we demonstrated using both ex vivo and in vivo models of CNV that GzmB can induce CNV by modifying ECM components in the outer retina. Although GzmB has established pro-fibrotic roles in dermal wound healing and cardiac fibrosis [11, 12], its role in subretinal fibrosis has yet to be determined. In the present study, we used the two-stage laser-induced mouse model of subretinal fibrosis to compare GzmB-deficient mice to WT mice, demonstrating for the first time that GzmB contributes to the early vascularized stage of subretinal fibrotic lesion development.

### Role of aging on subretinal fibrosis

Age is one of the most consistent risk factors for nAMD [34], and we previously reported that extracellular GzmB accumulation in the outer retina increases in an age-dependent manner in mouse, non-human primate and human eyes [5, 9]. This is consistent with a recent study in which GzmB was identified as a predictive plasma protein marker of mortality and risk of common age-related diseases in diverse populations [35]. Furthermore, human eyes with CNV show more GzmB immunoreactive cells in the choroid than age-matched control eyes [3, 9]. This suggests that extracellular GzmB in the outer retina could be an age-related risk factor for nAMD.

In support of this, we found that: (1) older WT mice had much larger subretinal fibrotic lesions than younger WT mice, and (2) GzmB deficiency resulted in significantly smaller subretinal fibrotic lesions, compared to WT, in older mice (Fig. 2A-F). These findings suggest that age-dependent accumulation of extracellular GzmB in the outer retina may contribute to subretinal fibrosis. Sex-based differences were observed in WT mice, reflecting cohort composition, as most of the older WT mice were male. However, no such differences were seen in GzmB KO mice (Fig. S2C-D). A limitation of this study is the unequal numbers of male and female mice in the age cohorts; hence, future work is needed to investigate the role of sex in the development of subretinal fibrosis in aged mice.

In vivo imaging analyses further revealed distinct lesion morphologies between genotypes. OCTA and DOPU imaging showed that WT mice exhibited broader lateral deformation, while GzmB KO mice displayed elevated axial lesions with reduced lateral spread (Fig. 2A–B). Interestingly, although fibrotic lesions generated using the Micron IV laser system exhibited weak CD31 immunoreactivity (Fig. S1B), suggesting a lack of ongoing neovascularization, OCTA imaging revealed vascular signals in these fibrotic lesions. While previous studies have employed alternative laser systems and parameters to induce fibrovascular membranes with strong CD31 immunolabeling [14, 36], our use of the Micron IV system with settings similar to those previously optimized for CNV induction using the same system [37] provides a distinct yet complementary approach, resulting in fibrotic lesions with residual vasculature but without active CNV. This also supports the notion that subretinal fibrosis in this model arises secondary to CNV but progresses independently of sustained neovascular activity. Indeed, prior work has shown that intravitreal injection of aflibercept, a VEGF inhibitor, immediately after the second laser fails to attenuate subretinal fibrosis, suggesting that fibrosis in this model is not fully dependent on ongoing CNV or VEGF activity [38].

Taken together, these findings suggest that GzmB contributes to age-dependent subretinal fibrosis by promoting lesion expansion and proteolytic disruption of extracellular matrix integrity. GzmB deficiency may attenuate these processes by limiting extracellular GzmB buildup and disrupting molecular events involved in the angiofibrotic switch, including fragmentation of key ECM proteins involved in nAMD, particularly TSP-1 and DCN.

### Cleavage of TSP-1 and DCN by GzmB contributes to subretinal fibrosis

To investigate how GzmB deficiency suppresses subretinal fibrosis, we examined the expression of two GzmB substrates, TSP-1 and DCN, within subretinal fibrotic lesions in WT and GzmB KO mice. TSP-1 exerts anti-angiogenic effects against choroidal sprouting, and GzmB promotes choroidal sprouting by cleaving TSP-1 in the outer retina. In line with this, human eyes with CNV showed reduced TSP-1 and elevated GzmB immunoreactivity in the outer retina, suggesting that GzmB-mediated fragmentation of TSP-1 may contribute to CNV [3].

Unexpectedly, TSP-1 immunolabeling within subretinal fibrotic lesions was lower in GzmB KO mice, compared to WT mice (Fig. 3A, C), contrary to our expectation that GzmB deficiency would preserve TSP-1 and enhance its detection. However, myofibroblasts are known to highly express TSP-1 in vitro and in vivo [39, 40], and our ARPE-19 culture data also indicated that acute GzmB stimulation results in TSP-1+ α-SMA+ cells (Fig. S7B, C). This suggests that elevated TSP-1 immunolabeling in the fibrotic lesions in WT mice may be partially attributable to GzmB’s proteolytic activity on RPE cells.

As for the decreased TSP-1 immunolabeling within subretinal fibrotic lesions in GzmB KO mice, there are two possible explanations. The first possible explanation is that GzmB deficiency may have prevented GzmB’s proteolytic activity on RPE cells, thereby reducing the overall TSP-1 expression in these lesions. The second possible explanation is that GzmB deficiency might have inhibited the fragmentation of both TSP-1 and DCN. This could have facilitated greater interaction between full-length TSP-1 and DCN within these lesions. Notably, DCN binds to the N-terminal domain of TSP-1 [41, 42], which contains the target epitope for the TSP-1 antibody (Abcam, ab26390) used in this study. Hence, DCN may have masked this epitope, preventing effective labeling of TSP-1 in subretinal fibrotic lesions in GzmB KO mice.

This masking may have also attenuated subretinal fibrosis in GzmB KO mice, as the N-terminal domain of TSP-1 can interact with calreticulin and stimulate fibroblasts to produce and secrete Col1 [19]. Although DCN is not known to bind to TSP-1 type-1 repeats and inhibit their capacity to activate latent TGF-β, it can bind to TGF-β and suppress TGF-β-mediated fibrosis [43]. Furthermore, GzmB can release active TGF-β that is sequestered by DCN [44]. Hence, GzmB deficiency may suppress subretinal fibrosis by preventing GzmB-mediated DCN degradation and thus the release of active TGF-β from DCN. Consistent with this hypothesis, fibrotic lesions in GzmB KO mice exhibited increased DCN immunolabeling compared to WT mice (Fig. 3B, D).

DCN+ myofibroblasts were present in both WT and GzmB KO mice (Fig. 3B), consistent with known DCN expression in these cells [45]. Notably, DCN+ α-SMA- cells were observed only in the fibrotic lesions in GzmB KO mice, potentially representing fibroblasts or choroidal endothelial cells [46]. Further studies are required to clarify their identity and role in subretinal fibrosis.

### GzmB contributes to chronic inflammation in subretinal fibrotic lesions and photoreceptor cell death

**Fig. 10:**
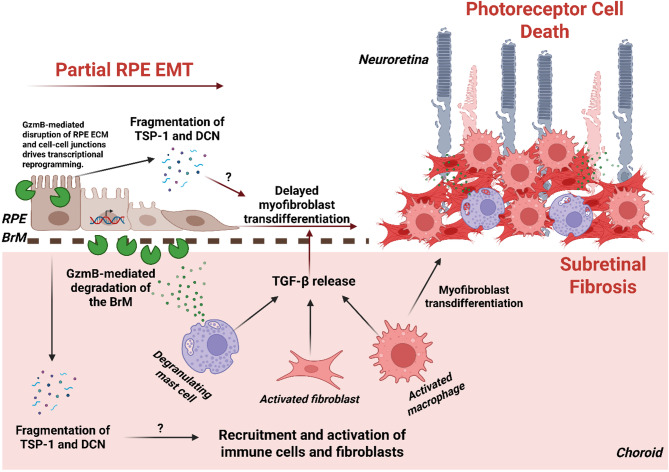
The role of GzmB in the pathogenesis of subretinal fibrosis in neovascular age-related macular degeneration. During the angiofibrotic switch in nAMD, choroidal mast cells degranulate their products, including GzmB. GzmB degrades the Bruch’s membrane (BrM), tight junctions and extracellular matrix of RPE cells. GzmB’s proteolytic activity leads to partial RPE EMT and delayed myofibroblast transdifferentiation in response to TGF-β released from various cellular sources, such as mast cells, fibroblasts and macrophages. GzmB-mediated fragmentation of TSP-1 and DCN may contribute to transdifferentiation of RPE cells to myofibroblasts and recruitment of immune cells and fibroblasts. Within the subretinal fibrotic lesion, mast cells continue to degranulate inflammatory mediators, including tryptase, and macrophages undergo MMT and transdifferentiate into myofibroblasts. The GzmB-mediated development of subretinal fibrotic lesion leads to photoreceptor death (created with BioRender.com)

Extracellular GzmB can promote chronic inflammation, as it can generate ECM fragments that function as chemoattractants for immune cells and extend inflammation [5, 11]. In the outer retina, GzmB can promote fragmentation of TSP-1 and DCN and prevent their anti-angiogenic activities, ultimately resulting in CNV [5]. Additionally, there is evidence that fragmentation of TSP-1 and DCN may promote the recruitment of immune cells and prolong inflammation; for instance, the C-terminal fragment of TSP-1 is chemotactic for immune cells [47] while DCN fragments can function as damage-associated molecular patterns (DAMPs) that can trigger inflammation [48, 49] (Fig. 10).

Since GzmB deficiency may result in reduced fragmentation of TSP-1 and DCN within subretinal fibrotic lesions, we expected that it will result in reduced accumulation of immune cells within subretinal fibrotic lesions. Surprisingly, GzmB deficiency increased the accumulation of macrophages within subretinal fibrotic lesions and promoted MMT (Fig. 4A-E). However, given that there were more CD206+ α-SMA+ cells within subretinal fibrotic lesions and in the choroid below the lesions in GzmB deficient mice (Fig. 4F), GzmB deficiency may have facilitated the M1-to-M2 phenotypic shift that happens as part of the natural wound healing process [50]. In support of this idea, GzmB deficiency has been shown to result in facilitated wound healing in the skin [12]. By preserving the integrity of TSP-1 and DCN, GzmB deficiency may create a less pro-inflammatory environment wherein the M1-to-M2 phenotypic shift happens more efficiently.

Additionally, we confirmed, for the first time, accumulation and degranulation of mast cells within subretinal fibrotic lesions and found that GzmB deficiency prevented accumulation of mast cells within subretinal fibrotic lesions (Fig. 5A-C). While the mechanisms by which mast cells influence macrophage polarization remain unclear, mast cells have been associated with M1 macrophages. For instance, the accumulation of mast cells is associated with a predominance of M1 macrophages in ruptured aneurysms [51] and obesity-related glomerulopathy [52]. Hence, GzmB deficiency may have prevented the accumulation of mast cells, facilitating the M1-to-M2 phenotypic shift and contributing to wound resolution. However, further study is needed to define the exact role of CD206+ α-SMA+ M2 macrophages in subretinal fibrosis. GzmB deficiency may have also influenced the functions of other immune sources of extracellular GzmB, such as neutrophils, T-cells and natural killer (NK) cells. Recent evidence suggests that neutrophils contribute to the development of CNV by forming neutrophil extracellular trap, stimulating RPE cells to release pro-inflammatory cytokines and promoting cell cycle arrest in RPE cells [53, 54]. On the contrary, both **γδ**T-cells and NK cells were shown to exert a protective role against the development of CNV [55, 56]. It remains unclear how GzmB deficiency would influence these immune subsets in the context of subretinal fibrosis. However, GzmB deficiency may reduce the cytotoxic potential of these cells and possibly shift their roles towards tissue repair. For example, GzmB deficient CD4+ T cells exhibit increased IL-17 production [57], and this may contribute to suppression of subretinal fibrosis, as IL-17 has been shown to suppress the development of tubulointerstitial fibrosis in the kidney [58]. However, further studies are required to determine how GzmB deficiency alters the functions of GzmB+ immune cells in the context of nAMD.

The presence of M2 macrophages, together with reduced accumulation of mast cells, was associated with decreased photoreceptor cell death in GzmB deficient mice. Because mast cells play a critical role in chronic inflammation and fibrosis [31], reduced mast cell accumulation within subretinal fibrotic lesions in GzmB KO mice may have resulted in a more favorable microenvironment for photoreceptor cell survival. Additionally, GzmB has been shown to directly induce neurotoxicity *via* activation of protease-activated receptor 1 (PAR1) [59]. Since photoreceptors express PAR1 [60], it is possible that GzmB released from immune cells within subretinal fibrotic lesions, such as choroidal mast cells, activates PAR1 and leads to photoreceptor cell death. Hence, GzmB deficiency may have limited this PAR1-dependent neuronal death pathway and reduced photoreceptor cell death.

Macroglia are generally known to maintain neuronal survival in the retina by providing neurotrophic factors and antioxidants [61]. However, the exact role of macroglia is largely unknown in the context of subretinal fibrosis in nAMD. Given that GFAP immunoreactivity in the ONL above subretinal fibrotic lesions was similar between WT and GzmB KO mice, GzmB deficiency does not seem to influence the activation of macroglia. GzmB deficiency may have led to a phenotypic shift towards neuroprotective A2-like macroglia [61], considering reduced photoreceptor cell death in GzmB KO mice. However, further study is required to confirm if and how GzmB can influence phenotypes of macroglia.

### GzmB impairs wound closure in the RPE by promoting partial epithelial-mesenchymal transition

The RPE serves as a major component of the outer blood-retinal barrier between the neuroretina and the choroid, protecting the neuroretina from blood-borne toxins and pathogens [62]. Aging, a major risk factor for nAMD, is believed to cause the RPE barrier dysfunction and ultimately contribute to the pathogenesis of nAMD [9, 62]. Previously, we have shown that extracellular GzmB accumulates in the outer retina in an age-dependent manner across rodent, non-human primate and human eyes, suggesting that this is an age-related pathological process leading to the pathogenesis of nAMD [9]. Indeed, we have demonstrated that exogenous GzmB not only compromises the RPE integrity by cleaving tight junction, cell adhesion and ECM proteins but also induces choroidal sprouting in the RPE-choroid explants [3, 9, 15].

Currently, there is no well-established in vitro model of subretinal fibrosis. However, the ARPE-19 wound healing assay provides a valuable tool for studying two key processes involved in fibrosis: EMT and wound closure. EMT is a key process involved in fibrosis wherein epithelial cells stop maintaining their cell-to-cell contact, lose their epithelial characteristics and eventually acquire mesenchymal characteristics, such as increased motility and contractility [63]. RPE cells are known to undergo EMT upon loss of their tight junctions and subsequent changes that initiate the development of subretinal fibrosis [4, 64]. Given that GzmB cleaves tight junction, cell adhesion and ECM proteins, we initially hypothesized that it would promote RPE EMT and facilitate wound closure. However, our findings revealed an unexpected role for GzmB in delaying wound closure in ARPE-19, suggesting that it impairs the normal wound healing in RPE cells. This reinforces the idea that GzmB plays a pathological role in subretinal fibrosis by disrupting normal RPE wound healing and allowing larger fibrotic lesions to form. Indeed, WT mice exhibited significantly larger subretinal fibrotic lesions, compared to GzmB KO mice (Fig. 2A-F). In addition, GzmB resulted in a mixed pattern of wound expansion and closure in the presence of TGF-β2, indicating that it may interfere with TGF-β2-mediated wound healing pathways. Interestingly, the sequential administration (6-hour GzmB stimulation → TGF-β2 treatment up to 72 h) eliminated the wound expansion, suggesting that the wound expansion is caused by the presence of both GzmB and TGF-β2. This indicates a complex interplay between GzmB and TGF-β2 that leads to impaired wound closure. In line with this idea, our RNA sequencing data revealed that acute GzmB stimulation leads to significant downregulation of genes involved in TGF-β signaling and EMT. Surprisingly, acute GzmB stimulation simultaneously leads to significant upregulation of certain EMT-related genes and inflammatory genes. These findings together suggest that while GzmB’s proteolytic activity may prevent RPE cells from responding effectively to TGF-β2, it may simultaneously activate alternative pathways that drive EMT and inflammatory responses. Hence, we hypothesize that GzmB’s proteolytic activity promotes partial EMT in RPE cells, given that TGF-β signaling is a major driver of full EMT [65].

In support of this, we observed significant transcriptional downregulation of TJP1 (ZO-1), PCDH1 and TNS1 alongside significant transcriptional upregulation of GJA1 and DSC1 (Fig. 8B). The transcriptional upregulation of GJA1 is particularly notable and aligns with prior evidence that its transcript levels increase while protein levels concurrently decline during EMT [66]. Together, these changes highlight how GzmB’s proteolytic activity modulates gene expression of specific tight junction, gap junction and cell adhesion proteins, contributing to the partial EMT phenotype. Further qPCR analysis of key genes highlighted in the heatmap revealed that GzmB’s proteolytic activity leads to significant upregulation of EMT- and inflammation-related genes (e.g. BMP4, IL-33 and GJA1) and downregulation of angiogenic genes (e.g. VEGF-A) at 18 h (Fig. S8). These findings suggest that GzmB-mediated transcriptional changes may start as early as 6 h, but become more pronounced by 18 h. While most RNA sequencing trends were supported by qPCR, SOX4, an EMT-related transcription factor [67], was significantly downregulated in the RNA sequencing dataset but showed no change by qPCR. This highlights gene-specific variability and the importance of validating transcriptomic findings across platforms. The delayed accumulation of mRNA for several targets suggests progressive or secondary transcriptional activation, reflecting a time-dependent regulatory effect exerted by GzmB’s proteolytic activity on RPE cells.

Our immunocytochemical data provide further evidence supporting partial EMT in RPE cells following GzmB stimulation. In the wound healing assay, ARPE-19 cells at the wound edges exposed to GzmB for 48 h exhibited a heterogeneous pattern of β-catenin localization, including membrane-bound, cytoplasmic, and nuclear staining (Fig. S6). This suggests that GzmB’s proteolytic activity disrupts TGF-β and/or Wnt signaling pathways and alters β-catenin activation dynamics. This aberrant localization implies that GzmB may impair wound closure by interfering with RPE cell migration and proliferation. Notably, only GzmB-stimulated cells exhibited long α-SMA+ fibers at the wound edges, further supporting the notion that GzmB promotes partial EMT. In this state, RPE cells may initiate EMT-related changes but remain incompletely differentiated and functionally immobile, which could contribute to delayed wound healing. This impaired repair response may explain the larger subretinal fibrotic lesions observed in WT mice compared to GzmB KO mice (Fig. 2). Based on this notion, we speculate that GzmB plays a pathological role in driving fibrotic lesion expansion in vivo.

To further investigate the effects of GzmB on RPE cells independent of mechanical injury, we performed immunocytochemistry on intact ARPE-19 monolayers without wounding. Following acute GzmB stimulation, we observed clusters of TSP-1+ α-SMA+ cells, indicating the initiation of mesenchymal characteristics (Fig. S7A-C). However, when these cells were subsequently exposed to TGF-β2 for 48 h, we observed clusters of elongated cells expressing EMT markers, including α-SMA, TSP-1, Col1 and fibronectin, along with a loss of tight junction protein ZO-1 (Fig. 9A-C). While these elongated cells resembled myofibroblasts, they did not fully acquire organized, bundled α-SMA stress fibers characteristic of fully differentiated myofibroblasts observed in subretinal fibrotic lesions (Fig. 1C). This suggests that GzmB’s proteolytic activity promotes partial RPE EMT through downregulation of genes involved in TGF-β signaling. We speculate that the GzmB-mediated partial RPE EMT delays wound closure and thereby contributes to prolonged duration of subretinal fibrosis in nAMD.

### Overlapping and differential roles of GzmB in CNV and subretinal fibrosis

Previously, we have established that GzmB promotes CNV by (1) cleaving anti-angiogenic ECM proteins TSP-1 and DCN in the outer retina and (2) releasing pro-angiogenic growth factors TGF-β and VEGF-A sequestered in the outer retinal ECM [3, 15]. Although these molecular events are directly involved in CNV, they can also contribute to subretinal fibrosis. Fragmentation of TSP-1 and DCN can act as pro-fibrotic factors or DAMPs, prolong inflammation and contribute to fibrotic remodeling [19, 48]. TGF-β is a well-known driver of fibrosis that can induce both EMT and MMT [34]. Hence, some of the pro-angiogenic mechanisms driven by GzmB may also contribute to subretinal fibrosis, and this overlap may underlie the angiofibrotic switch in nAMD.

Our data indicate that GzmB may promote pro-fibrotic mechanisms via RPE cells through transcriptional regulation. As discussed above, GzmB impairs RPE cell migration and proliferation and delays RPE wound closure by downregulating key genes involved in the TGF-β signaling pathway. In parallel, GzmB induces a mixed pattern of up/downregulation of genes involved in cell-cell interaction, along with increased expression of EMT protein markers in RPE cells. By delaying full EMT, GzmB likely impedes myofibroblast transdifferentiation, a process required for wound contraction and resolution [68]. Additionally, GzmB upregulates pro-inflammatory genes in RPE cells that may directly contribute to subretinal fibrosis; for instance, GzmB-mediated transcriptional upregulation of IL-33 and PTX3 in RPE cells may lead to their production and release and trigger the activation of mast cells and amplify ongoing inflammation in the outer retina [69, 70]. Simultaneously, GzmB downregulates key pro-angiogenic genes, such as VEGF-A and PDGF-A, in RPE cells (Fig. 8B and Fig. S8D). This suggests that GzmB may suppress angiogenic responses while promoting transcriptional and phenotypic changes in RPE cells indicative of partial EMT. Together, these findings suggest that GzmB has both overlapping and differential roles in CNV and subretinal fibrosis.

Given that GzmB stimulation downregulates genes involved in TGF-β signaling and angiogenesis in ARPE-19 (Fig. 8) but also results in increased release of TGF-β and VEGF-A in the supernatant of outer retina explants [15], GzmB’s proteolytic activity may prime other cell types in the choroid, such as mast cells, macrophages, fibroblasts and endothelial cells, to produce and release TGF-β isoforms and VEGF-A. These cells then may together promote CNV and progressively push RPE cells towards myofibroblast transdifferentiation via TGF-β signaling. GzmB may also modify the RPE ECM and its key components, such as TSP-1 and DCN, and induce myofibroblast transdifferentiation in RPE cells (Fig. 10). However, further investigation is needed to determine the role of GzmB-mediated fragmentation of TSP-1 and DCN in RPE EMT and myofibroblast transdifferentiation.

In conclusion, we demonstrated multiple features associated with the novel role of GzmB in the pathogenesis of subretinal fibrosis in nAMD (Fig. 10). Building on our previous findings regarding the role of GzmB in CNV, we propose that GzmB contributes to the angiofibrotic switch by promoting both neovascular and fibrotic components of subretinal lesion formation in nAMD. Pharmacologically targeting extracellular GzmB activity or its release from mast cells may represent a promising therapeutic approach, either as an alternative or adjuvant to anti-VEGF therapy, for patients with nAMD who are refractory or non-responsive to current treatments. Future research should focus on developing specific inhibitors of GzmB and/or mast cells to suppress both CNV and subretinal fibrosis.

## Methods

### Animals

All animal experiments adhered to the Canadian Council on Animal Care guidelines and were approved by the University of British Columbia. Both male and female adult C57BL/6J (WT) and GzmB KO mice on a C57BL/6J background were obtained from the Jackson Laboratory (Bar Harbor, ME, USA). Mice were grouped by age: younger (3–6-month) and older (7–14-month) groups. All mice were housed with consistent light-dark cycle and with food and water provided.

### Two-stage laser model of subretinal fibrosis

Subretinal fibrosis was induced in WT and GzmB KO mice following published protocols for the two-stage laser-induced mouse model of subretinal fibrosis [14, 71]. Mice were anesthetized with isoflurane, and pupils were dilated using 2.5% Mydfrin and 1% Mydriacyl (Alcon, Fort Worth, TX). Four separate laser burns were made at equal distance from the optic disc using an image-guided laser system (Micron IV, Phoenix Research Laboratories, Pleasanton, CA). The laser settings were 532 nm wavelength, 250 mW power, 100 ms duration, and 50 μm spot size. The first laser burns were applied on Day 0, and the second laser burns to the same sites on Day 7 to induce subretinal fibrosis (Fig. 1A). All eyes were enucleated on Day 14.

### In vivo imaging of lasered eyes

 In vivo functional retinal imaging was performed on Day 14 using a polarization-diversity optical coherence tomography (PD-OCT) specifically designed for small animals [18]. A superluminescent laser diode (BLM2-D, Superlum Diodes Inc., Ireland) with a central wavelength of 810 nm and 1/e^2^ bandwidth of 90 nm was used as a light source yielding the axial resolution of 3.2 μm in air. The imaging beam with a diameter of 1 mm and optical power was restricted to be less than 1 mW at the pupil. The backscattered beam from the subject was detected by two custom-built spectrometers that are numerically cross-calibrated [72, 73] to ensure the signals are identical with opposite polarity for PD detection. Structural OCT images were generated by averaging complex signals from both spectrometers to form polarization-insensitive coherent composite images, compensating for the signal loss due to channel separation. OCT-based angiography (OCTA) images were obtained from three consecutive OCT scans at a single transversal location to visualize retinal vasculature. Each OCT and OCTA volume comprised 500 A-scans per B-scan and 500 B-scans per volume, acquired at 242 kHz of A-scan and 440 Hz of B-scan rates, respectively. The depolarization contrast visualizing melanin component in the retina was derived by calculating the noise-suppressed degree of polarization uniformity (DOPU) [74]. All data processing and image reconstruction were performed using MATLAB-R2022b (Mathworks Inc., CA, USA).

### Ocular tissue processing

Mouse eyes were fixed in 10% buffered formalin for two days and either embedded in paraffin for 6 μm retina cross-sections or dissected for RPE-choroid wholemounts. Retina cross-sections were deparaffinized with alcohol washes before staining. RPE-choroid wholemounts were with four radial cuts without damaging fibrotic lesions around the optic disc. For toluidine blue staining, RPE-choroid wholemounts were cleared in 10% H_2_O_2_ for one hour at 60 °C.

### Histological staining

#### Masson’s trichrome staining

A trichrome staining kit (Abcam, AB150686) was used to detect collagen within subretinal fibrotic lesions. Deparaffinized retina cross-sections were stained with hematoxylin (1:1 Weigert’s A and Weigert’s B) for 3 min and then rinsed in running tap water for 2 min. Biebrich Scarlet/Acid Fuchsin Solution was applied for 15 min and then rinsed in distilled water. Subsequently, the samples were differentiated in Phosphomolybdic/Phosphotungstic Acid Solution for 15 min. Without rinsing, Aniline Blue Solution was applied for 10 min. After rinsing the samples in distilled water, 1% Acetic Acid Solution was applied for 5 min, dehydrated in alcohol, cleared in xylene, and mounted with Permount. The samples were imaged at 60X using a Nikon Eclipse 80i.

#### Toluidine blue staining

Cleared RPE-choroid wholemounts were stained in 0.1% toluidine blue (Abcam, AB146366) in distilled water at pH 2–2.5.5 for 10–15 min. The samples were then washed in acidic PBS (pH = 2–2.5.5), mounted with an aqueous mounting medium, and imaged at 40X using a Nikon Eclipse 80i. Mast cells appeared pink/purple, and they were up 20 μm in diameter.

### Immunohistochemistry and immunocytochemistry

#### Retina cross-sections

All antibodies and their sources are listed in Supplemental Table 1. Retina cross-sections underwent antigen retrieval by heating in citrate buffer for 10 min. The samples were then incubated with a solution of 3% normal goat serum (NGS) (Vector Laboratories, S-1000-20) and 0.3% Triton X-100 in PBS to block non-specific binding and permeabilize the samples. The samples were then stained with antibodies for TSP-1, DCN, IBA1 or CD206 for 3 h at room temperature. Next, the samples were washed in PBS and incubated in secondary antibodies matched to primary antibodies. Anti-α-SMA antibody was used to locate subretinal fibrotic lesions. TUNEL staining was performed using in situ cell death detection kit (Roche, 11684795910) and following the protocol provided by the company. Subsequently, the samples were stained with anti-GFAP antibody for 2 h and then with a matching secondary antibody. All cross-sections were counterstained with 4′,6-diamidino-2-phenylindole (DAPI) and mounted with Mowiol 4–88 (Millipore, 475904) and 1.5 mm coverslips. Negative control samples were run in parallel, but with the omission of the primary antibodies.

#### RPE-choroid wholemounts

Wholemounts were treated with proteinase K for antigen retrieval. The samples were incubated in a solution of 3% NGS and 3% Triton X-100 in PBS to prevent non-specific binding and permeabilize the samples. To visualize and quantify the area of subretinal fibrotic lesions, anti-collagen-1 (Col1) antibody was used. Myofibroblasts and fibronectin in the subretinal fibrotic lesions were visualized with antibodies for α-SMA and fibronectin. Mast cells in the subretinal fibrotic lesions were visualized with anti-tryptase antibody and fluorescein-Avidin D. The wholemounts were incubated with antibodies for Col1, fibronectin or tryptase antibody for 3 h or with anti-α-SMA antibody or fluorescein Avidin D for one hour. Next, wholemounts were washed in PBS and incubated in secondary antibodies matched to the primary antibodies. Wholemounts were counterstained with DAPI (1/500) to visualize clusters of cell nuclei that served as landmarks for subretinal fibrotic lesions. Wholemounts were mounted with Mowiol 4–88 and 1.5 mm coverslips.

### ARPE-19 cell culture

Low passage (p3-6) ARPE-19 cells were seeded and maintained until confluent in 2 mL complete culture medium containing Dulbecco’s modified Eagle Medium/F12, high glucose, 1% penicillin/streptomycin and 10% fetal bovine serum (FBS), and they were incubated at 37 °C in a humidified chamber with 5% CO_2_ [75]. For GzmB stimulation, cells were seeded in complete culture medium in 8-well chamber slides and cultured to confluency. Prior to GzmB stimulation, cells were cultured overnight in medium containing 2% FBS. Cells were treated with either 10 ng/mL TGF-β2 for 48 h or 50 nM recombinant human GzmB for 6 h and then 10 ng/mL TGF-β2 (PeproTech) for 48 h. Cells were serum-starved during stimulation with 50 nM recombinant human GzmB (Bon Opus) diluted in culture medium without FBS for 6 h. Cells were fixed in 4% paraformaldehyde (PFA) for one hour at 4 °C before undergoing immunocytochemistry.

### ARPE-19 wound healing assay

For the wound healing assay, ARPE-19 cells were seeded in a 96-well cell culture plate (Corning) and maintained until 100% confluent. Scratches were made with the head of a 100 µL pipette tip to create wounds in a straight line of a constant width. Subsequently, the cells were cultured in serum-free media (SFM) (control groups), TGF-β2 (positive control group), 50 nM recombinant human GzmB (Bon Opus), 50 nM GzmB + 25 µM VTI-1002 (viDA Therapeutics Inc.), 50 nM GzmB + 10 ng/mL TGF-β2 (GzmB + TGF-β2 group), 50 nM GzmB + 10 ng/mL TGF-β2 + VTI-1002, SFM for 6 h and then 10ng/mL TGF-β2 (SFM→TGF-β2), 50 nM GzmB for 6 h and then 10ng/mL TGF-β2 (GzmB→TGF-β2) or 50 nM GzmB + 25 µM VTI-1002 for 6 h and then 10ng/mL TGF-β2 (GzmB + VTI-1002→TGF-β2). Cells were imaged by the phase contrast microscope (Nikon Eclipse TS100) soon after the scratch (0 h) and 24, 48 and 72 h after the scratch. The wound areas were quantified by using ImageJ, and the wound closure rate was calculated by using the following formula:

$$Wound\:closure\:rate =\:\frac{[{Wound\:Area}_{0\:hr}-{Wound\:Area}_{t}]}{{Wound\:Area}_{0\:hr}}$$

where t = 24, 48 and 72 h after the scratch wound.

### Immunocytochemistry

ARPE-19 cells in the chamber slides were blocked and permeabilized in 3% normal goat serum and 0.1% TX-100 PBS for 30 min before being immunolabeled with primary antibodies for α-SMA, ZO-1, TSP-1, Col1 and fibronectin for 2 h at room temperature. Next, the cells were stained with secondary antibodies matched to the primary antibodies and counterstained with DAPI (1/500) for 10 min. The chamber slides were mounted using Mowiol 4–88 and 1.5 mm coverslips. Negative control samples were run in parallel, but with the omission of the primary antibodies.

### Confocal microscopy

Retina cross-sections, wholemounts and chamber slides were imaged using a Zeiss LSM 800 confocal microscope with Zen 2.6 Blue version software (Carl Zeiss, Germany). Confocal microscopy was completed at 20X or 40X magnification. For retina sections and RPE-choroid wholemounts, Z-stack images were taken to create orthogonal reconstructions and capture all the fluorescent signals in each subretinal fibrotic lesion. For chamber slides, single-plane images were taken. Z-stack images were taken only in the cases in which there was a thick cluster of α-SMA+ cells on top of the monolayer of ARPE-19 cells. All confocal settings were kept constant throughout the imaging sessions to allow comparison of the intensity of fluorescent signals between WT and GzmB KO mouse eyes or between control (10 ng/mL TGF-β2 for 48 h) and GzmB (50 nM GzmB for 6 h and then 10 ng/mL TGF-β2 for 48 h) cell culture groups.

### Image analysis

For the areal measurements of the Col1 + subretinal fibrotic lesions in RPE-choroid wholemounts, the Col1 + fibrotic lesion was outlined, and measurements were calculated using ImageJ software. The immunofluorescence of TSP-1 or DCN was quantified by counting the number of TSP-1+ or DCN+ pixels within each α-SMA+ subretinal fibrotic lesion in retina cross-sections. The area of each α-SMA+ subretinal fibrotic lesion in retina cross-sections was measured and used for calculating the number of TSP-1+ or DCN+ pixels per mm^2^. The analysis of TSP-1 and DCN immunofluorescence was conducted independently by two blinded investigators.

The number of macrophages (IBA1+) or macrophages undergoing MMT (IBA1+ α-SMA+) within each α-SMA+ subretinal fibrotic lesion in retina cross-sections were manually counted. The area of α-SMA + subretinal fibrotic lesion in each retina cross-section was measured to estimate the IBA1+ or IBA1+ α-SMA+ cell density per mm^2^. The percentage of macrophages co-expressing α-SMA within a subretinal fibrotic lesion was calculated by dividing the IBA1+ α-SMA+ cell density by the IBA1+ cell density. The number of IBA1+ cells in the subretinal space right above each α-SMA+ subretinal fibrotic lesion was manually counted, and the length of the subretinal space was measured to estimate the number of IBA1+ cells per mm of the subretinal space. Cell counting was done independently by three blinded investigators.

Finally, the number of choroidal mast cells within each subretinal fibrotic lesion in RPE-choroid wholemounts was manually counted by a blinded investigator using fluorescein-Avidin D signals that was co-labeled with DAPI. The areas of subretinal fibrotic lesions containing mast cells were measured by highlighting the circumference of lesions on ImageJ software. Mast cell count in each subretinal fibrotic lesion was divided by the area of each subretinal fibrotic lesion to estimate the number of mast cells per mm^2^.

### Statistical analysis

GraphPad Prism (Version 10.3.1 GraphPad Software, San Diego, CA, USA) was used for performing Mann-Whitney U test or student’s t-test for comparing two groups and one-way ANOVA with post-hoc Tukey’s multiple comparison’s test for comparing more than two groups. All data are expressed as mean ± SEM, and *p* < 0.05 was considered statistically significant.

### ARPE-19 real time PCR

ARPE-19 cells were seeded in complete culture medium in 12-well plates and cultured to confluency. Prior to GzmB treatment, cells were cultured overnight in medium containing 2% FBS. Cells were serum-starved 30 min prior to stimulation with 100 nM recombinant human granzyme B for 6–18 h. After GzmB stimulation, ARPE-19 cells were lysed, followed by extraction of total RNA (Qiagen RNeasy kit) and reverse transcription using High Capacity cDNA Reverse Transcription kit (Applied Biosystems). Forward and reverse primers were designed and synthesized from Thermo Fisher Scientific for the genes of interest (BMP4, IL-33, VEGF-A, GJA1, SOX4). Quantitative PCR was performed using the QuantStudio 3 Real-Time PCR system (Thermo Fisher Scientific).

### ARPE-19 RNA bulk sequencing

ARPE-19 cells were seeded in complete culture medium in 12-well plates and cultured to confluency. Prior to treatment with GzmB, cells were cultured overnight in medium containing 2% FBS. Cells were serum-starved 30 min prior to stimulation with 100 nM recombinant human GzmB for 6 h. Total RNA was extracted from the cells after GzmB stimulation, with genomic DNA removal (Qiagen). Total RNA integrity was > 9, assessed with the TapeStation 4200 (Agilent Technologies; RNA Assay Cat No.5067–5576). RNA was quantified with the Qubit RNA HS assay (ThermoFisher; Cat No. Q32852). 100 to 200 ng of total RNA was used as input to enrich for mRNA with the NEBNext Poly(A) mRNA Magnetic Isolation Module (NEB; Cat No. E7490). Strand-specific libraries were prepared using the DNBSEQ Fast RNA Library Prep kit (Complete Genomics; Cat No. 940–001522−00). Library quality and size were checked with the TapeStation 4200 (Agilent Technologies; D1000 Assay Cat No. 5067–5582) and then quantified using the Qubit dsDNA HS Assay (ThermoFisher; Cat No. Q32854). The libraries were sequenced using a Complete Genomics DNBSEQ-G400RS High-throughput Sequencer to generate 30 million 100-bp paired-end reads per library.

Fastq files were aligned to the reference genome (hg38) with STAR (version 2.6.0a), and on-target reads are quality-controlled using MultiQC (10.1093/bioinformatics/btw354) that aggregates and visualizes quality control metrics from multiple bioinformatics and statistic tools. Reads were quantified with HTSEQ (version 0.11.2), and normalized/differential expression is calculated with DESeq2 (version 1.16.1). Statistics for group comparison include t-test or ANOVA, false discovery rate using the Benjamin-Hochberg multiple correction procedure with *p* < 0.05 and fold change of 2. DESeq2 used the Negative Binomial Generalized Linear Model (GLM) and shrinks the log fold-change (LFC) gene expression estimates. These shrunken LFCs overcame the issue of overestimating differences for weakly expressed genes. The Wald test was applied to the LFCs, and the resulting p-values were adjusted for multiple testing using the procedure of Benjamini and Hochberg.

## Supplementary Information

Supplementary Material 1.

## Data Availability

The authors declare that all the data supporting the findings of this study are available within the main text or the Supplemental Material.
