# Supplementary material for: Granzyme B contributes to subretinal fibrosis in neovascular age-related macular degeneration by modulating inflammation and epithelial-mesenchymal transition
Source: J Neuroinflammation. 2025 Nov 27;23:1. doi: 10.1186/s12974-025-03619-9 (PMC12764018; doi:10.1186/s12974-025-03619-9)
Supplement: Supplementary file 1 — Supplementary Material 1. [file 12974_2025_3619_MOESM1_ESM.pdf]

Supplementary Table 1: List of antibodies

Target	Reagent	Dilution	Source
α-SMA	Anti-alpha smooth muscle actin antibody (ab7817)	1/100	Abcam
CD206	Anti-CD206 antibody (AF2535)	1/100	R&D
Collagen-1	Anti-collagen I antibody	1/100	Abcam
DCN	Anti-Decorin antibody (ab175404)	1/50	Abcam
Fibronectin	Anti-fibronectin antibody (ab2413)	1/100	Abcam
GFAP	Anti-glial fibrillary acid protein antibody (Z0334)	1/100	Agilent Dako
IBA1	Anti-IBA1 antibody (019-19741)	1/100	Fujifilm Wako
Tryptase	Anti-tryptase antibody (MA5-38007)	1/150	Invitrogen
TSP-1	Anti-thrombospondin 1 antibody (ab263905)	1/100	Abcam
Alexa 488	Goat Anti-rabbit Alexa 488 secondary antibody (A11070)	1/500	Fisher Scientific
Alexa 488	Donkey Anti-goat Alexa 488 secondary antibody (A-11055)	1/500	Invitrogen
Alexa 546	Goat Anti-mouse Cy3 Alexa 546 IgG2a secondary antibody (A21133)	1/500	Fisher Scientific
Alexa 546	F(ab')2-Goat anti-Rabbit IgG (H+L) Cross-Adsorbed Secondary Antibody	1/500	Fisher Scientific

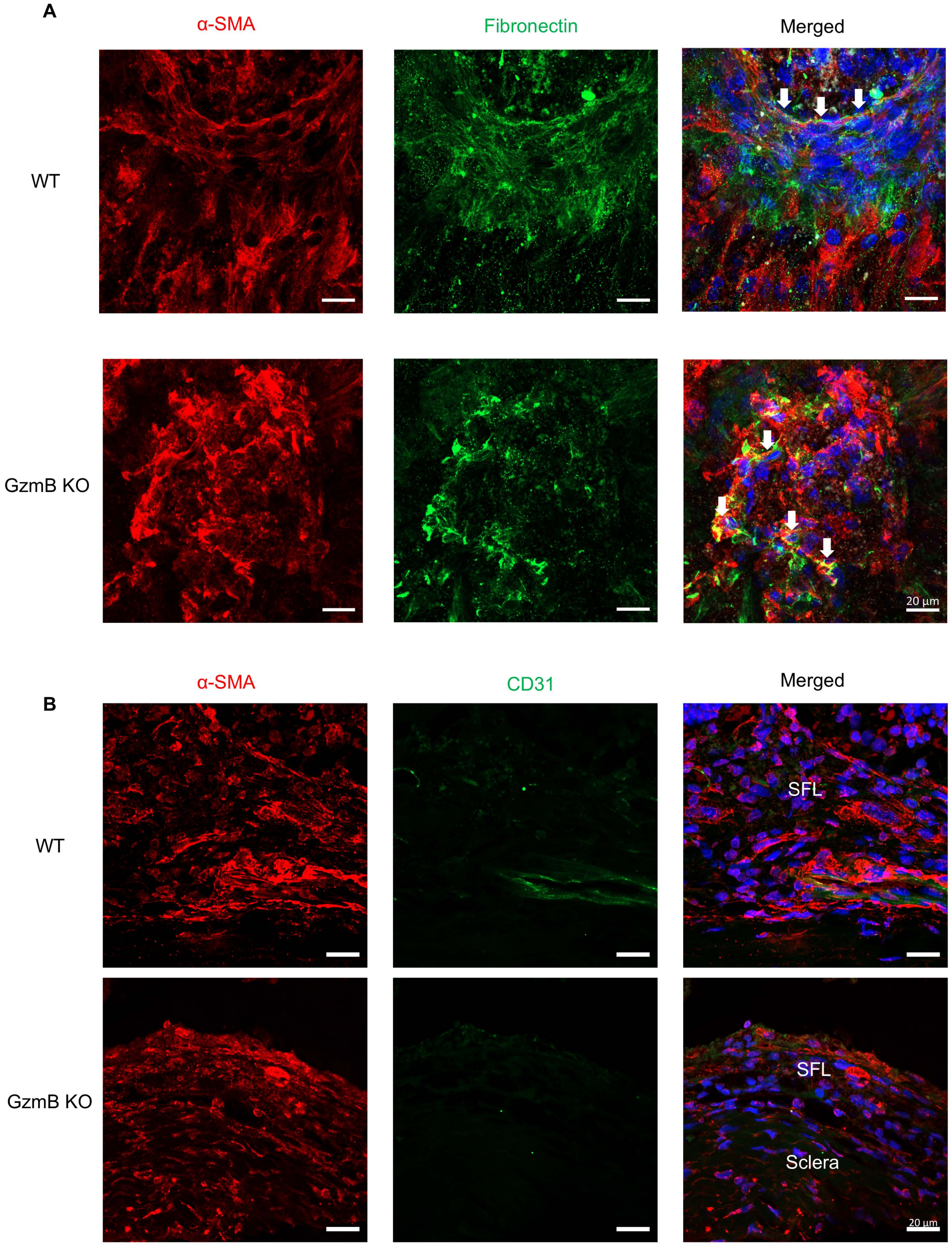

Supplemental Figure 1: Two-stage laser model with the Micron IV system (250 mW, 100 ms, 50 µm) results in fibrotic lesions consisting of myofibroblasts expressing fibronectin in both WT and GzmB KO mice without active endothelial component. A Representative 40X confocal images of subretinal fibrotic lesions consisting of α-SMA+ myofibroblasts (red) and fibronectin (green) in outer retina wholemounts from WT and GzmB KO mice. α-SMA+ fibronectin+ myofibroblasts are indicated by white arrows. Scale bars, 20 µm. **C** Representative 40X confocal images of subretinal fibrotic lesions with strong α-SMA (red) but weak CD31 (green) immunolabeling in retina cross-sections. Scale bars, 20 µm.

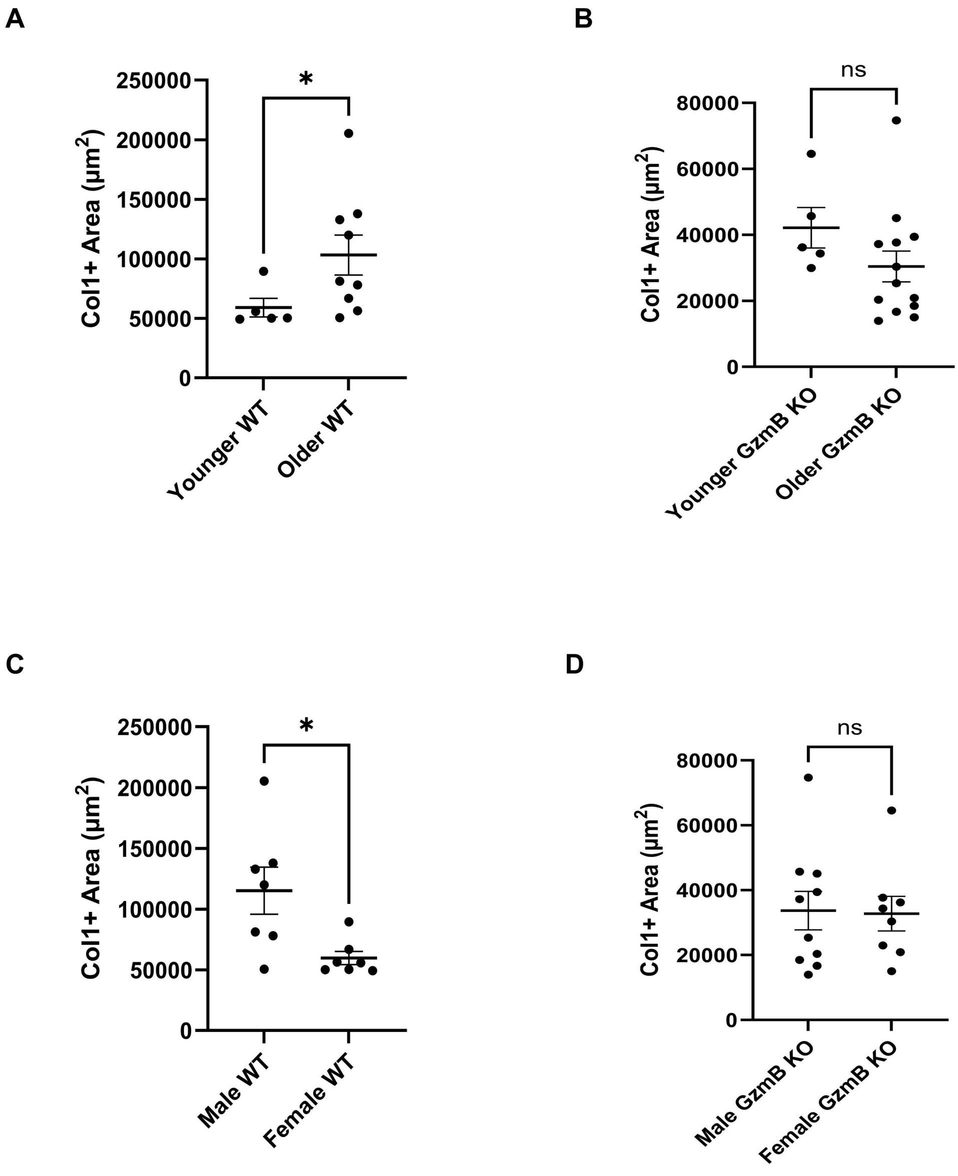

Supplemental Figure 2: Influence of age and sex on subretinal fibrosis in WT and GzmB KO mice. A, B Statistical analyses of the mean areas of Col1+ subretinal fibrotic lesions between younger (n = 5) and older (n = 9) WT mice (**A**) and between younger (n = 5) and older (n = 13) GzmB KO mice (**B**). **C, D** Statistical analyses of the mean areas of Col1+ subretinal fibrotic lesions between male (n = 7) and female (n = 7) WT mice (**C**) and between male (n = 10) and female (n = 8) GzmB KO mice (**D**). All statistical analyses were performed by Mann-Whiteny U test, *$p < 0.05$.

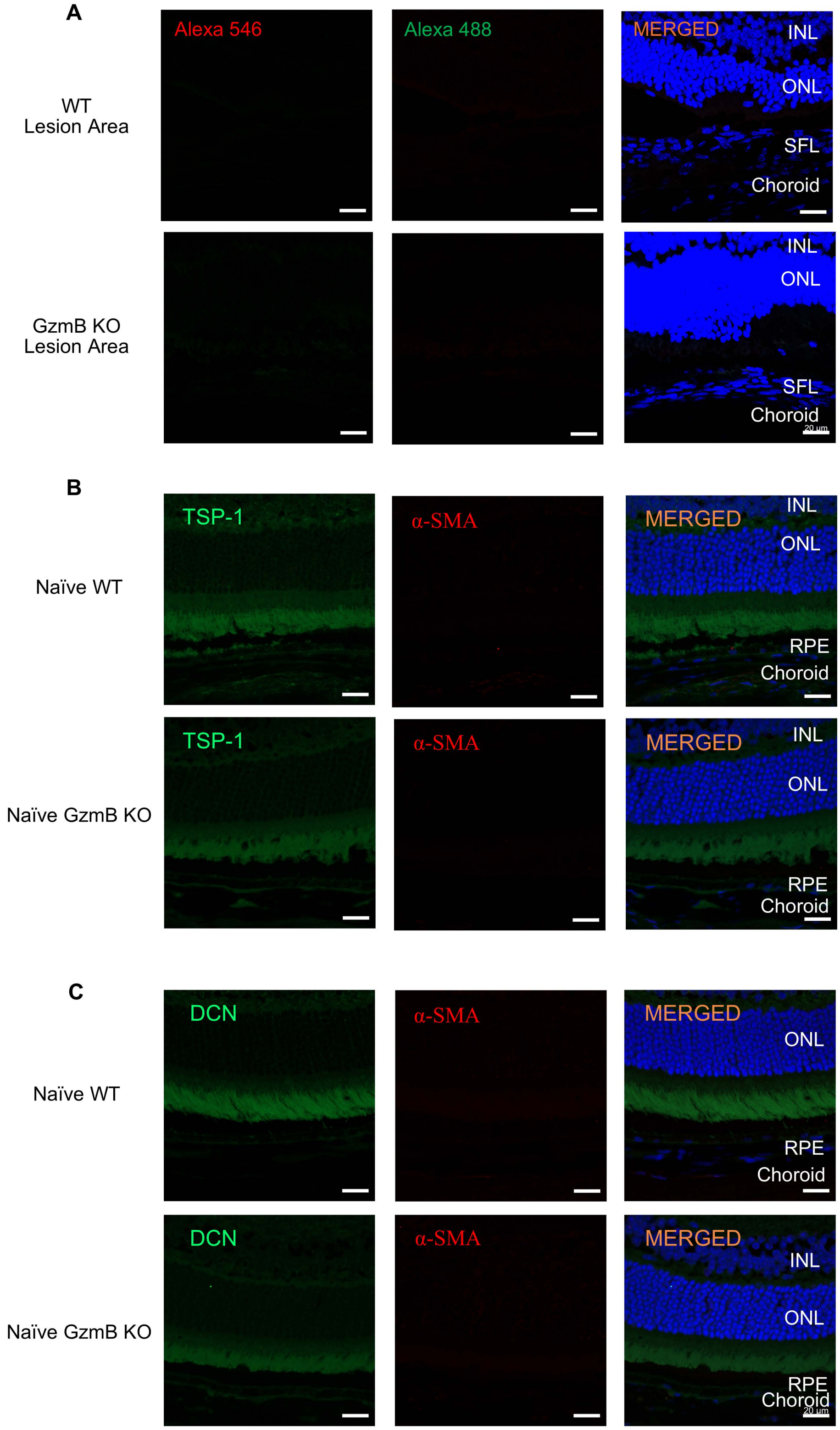

Supplemental Figure 3: Immunolabeling of TSP-1, DCN and α-SMA in naïve WT and GzmB KO mice. A Negative staining of retina cross-sections containing subretinal fibrotic lesions. **B, C** Immunolabeling of TSP-1 (**B**) or DCN (**C**) and α-SMA in naïve WT and GzmB KO mice. Scale bar, 20 μm. INL = Inner Nuclear Layer, ONL = Outer Nuclear Layer, SFL = Subretinal Fibrotic Lesion, RPE = Retinal Pigment Epithelium.

A

B

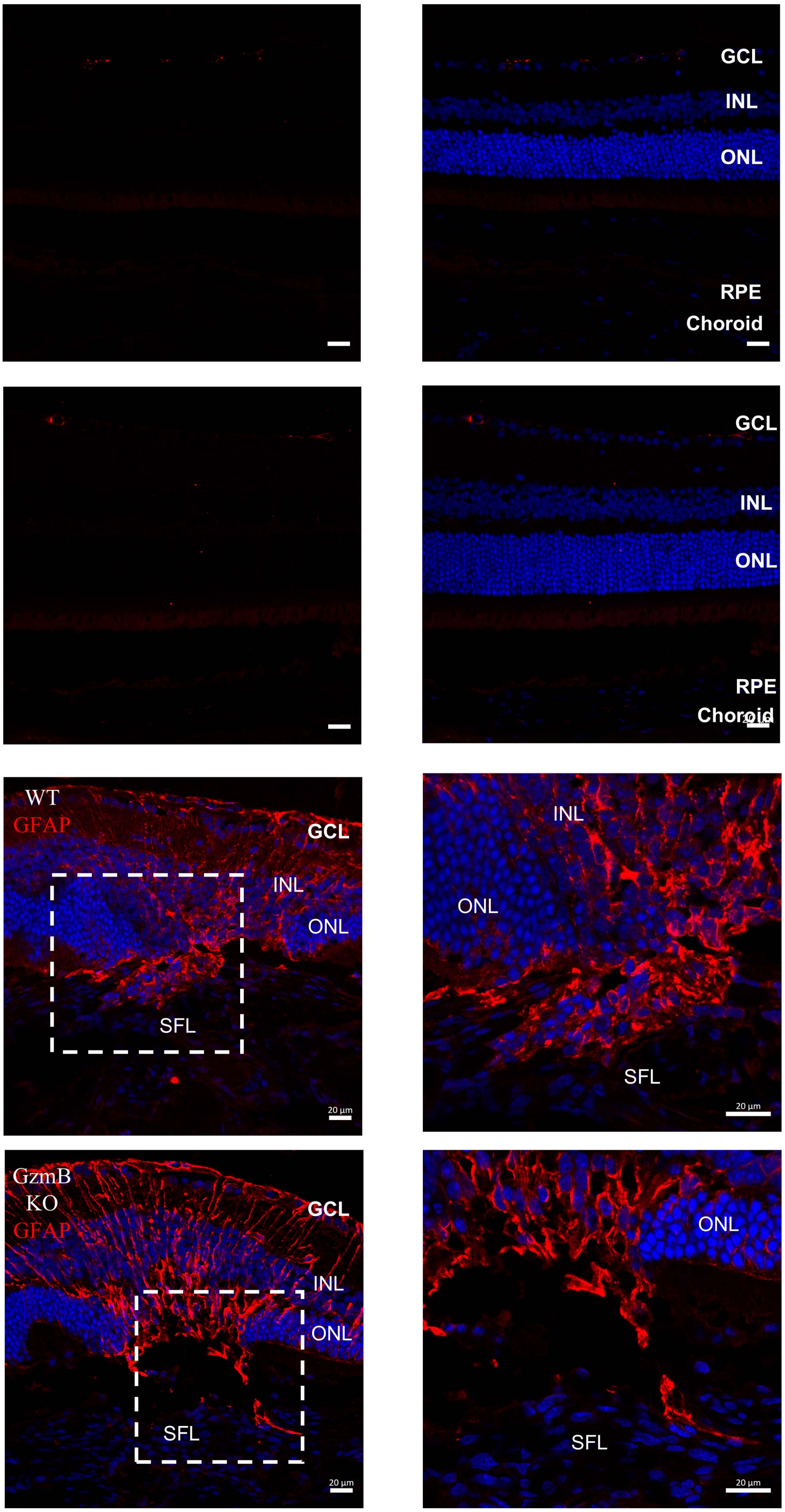

Supplemental Figure 4: GFAP+ cells located above subretinal fibrotic lesions in WT and GzmB KO mice. A Immunolabeling of GFAP (red) in the neuroretina in naïve WT and GzmB KO mice. **B** Immunolabeling of GFAP (red) in the neuroretina and subretinal fibrotic lesions in WT and GzmB KO mice**.** White dotted boxes indicate regions overlying SFLs, which are shown at 40X magnification in the corresponding right-hand panels. Nuclei are counterstained with DAPI (blue). GCL = Ganglion Cell Layer, INL = Inner Nuclear Layer, ONL = Outer Nuclear Layer, SFL = Subretinal Fibrotic Lesion.

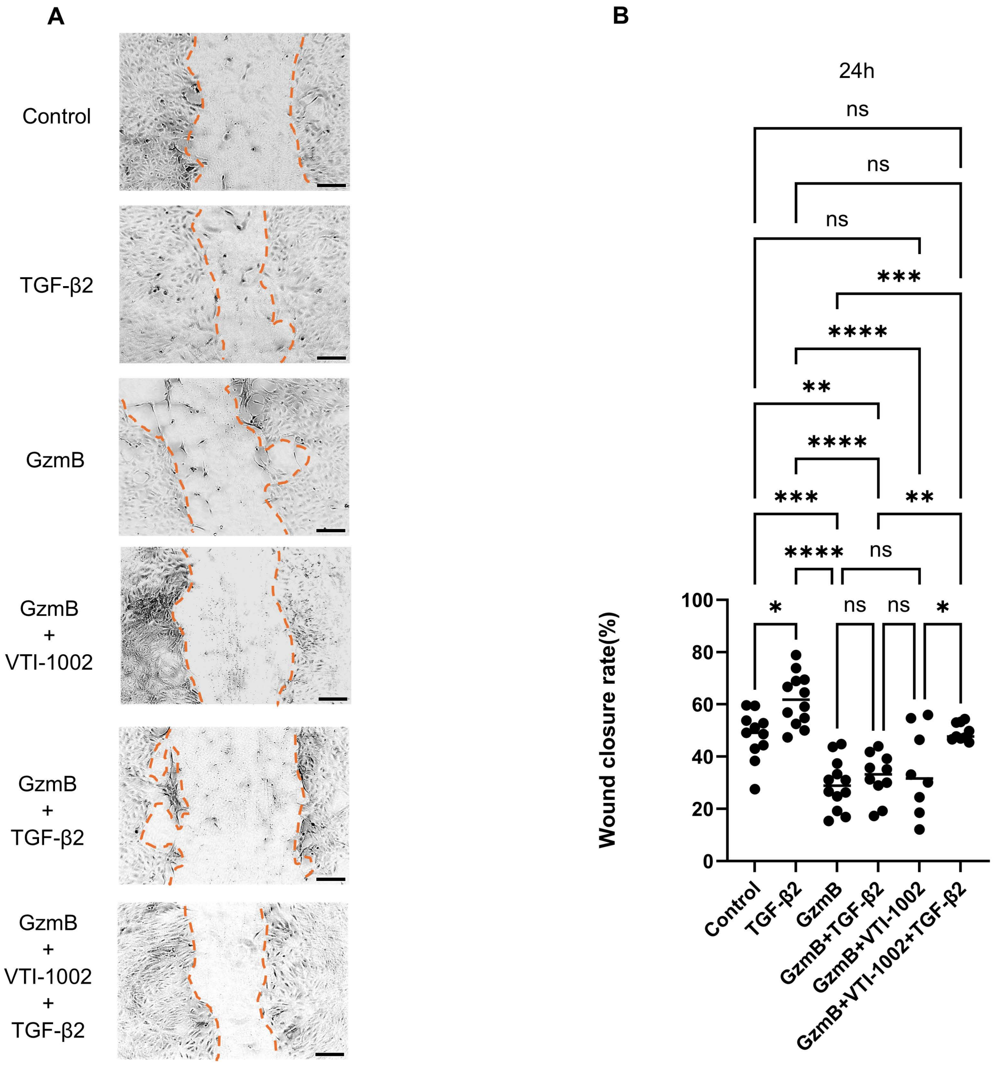

Supplemental Figure 5: GzmB delays wound closure in ARPE-19 at 24 hours. A Representative phase-contrast images of scratch wounds in each treatment group at a 24-hour timepoint. The borders of wound edges are indicated by orange dotted lines. Scale bar, 500 μm. **B** ARPE-19 wound closure rate in each treatment group at a 24-hour timepoint (n = 8-12 samples per group; representative of three-independent experiments). The data are presented as mean ± SEM. Statistical analyses were performed by one-way ANOVA Tukey's multiple comparison test, *$p < 0.05$, **$p < 0.01$, ***$p < 0.001$ and ****$p < 0.0001$.

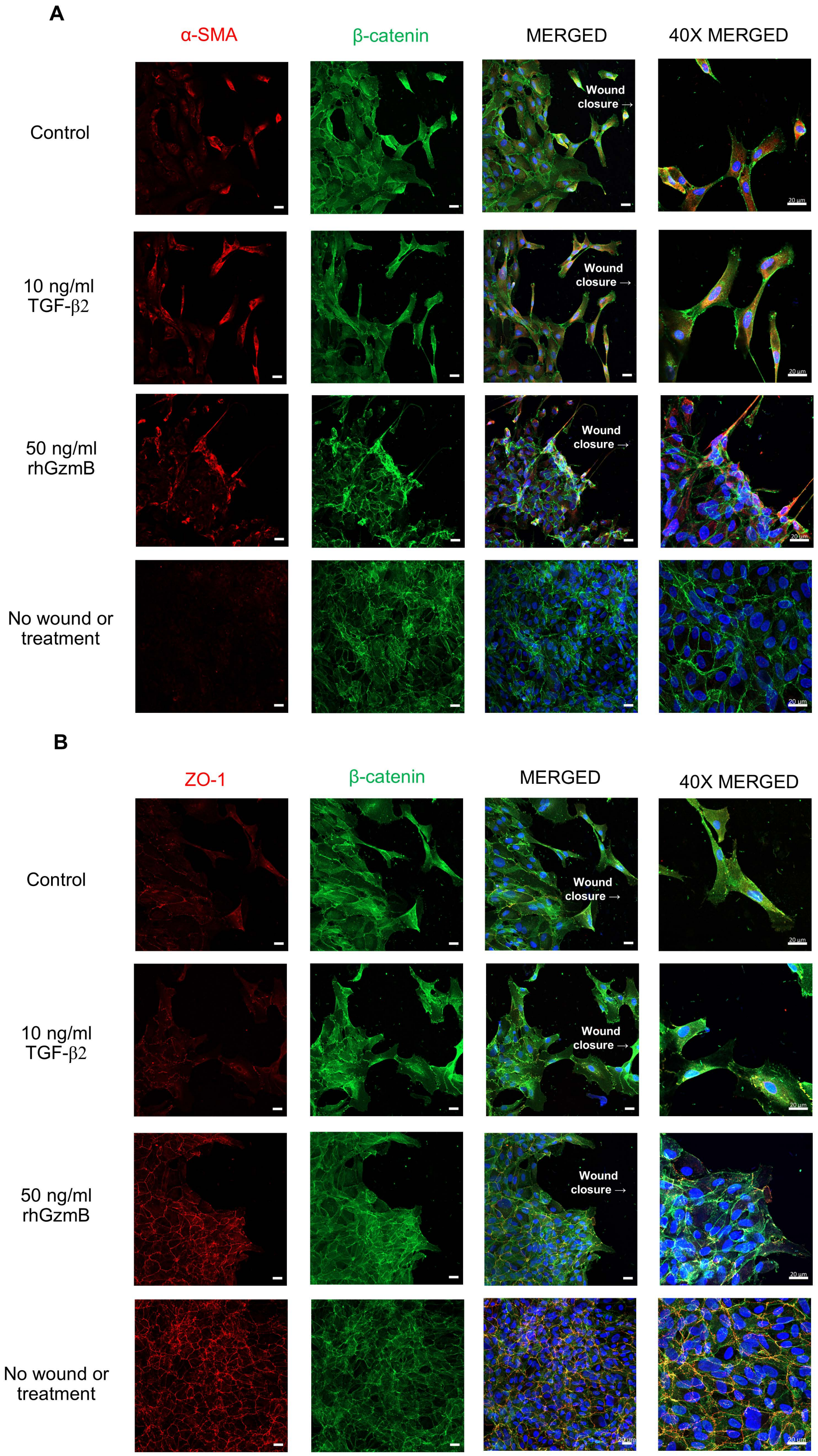

Supplemental Figure 6: GzmB impairs wound closure in ARPE-19 at 48 hours by modulating β-catenin localization. A, B Representative 20X and 40X confocal images of each treatment group showing α-SMA or ZO-1 and β-catenin expression at the wound edge of ARPE-19. The white arrows indicate the direction of wound closure. Scale bar, 20 μm.

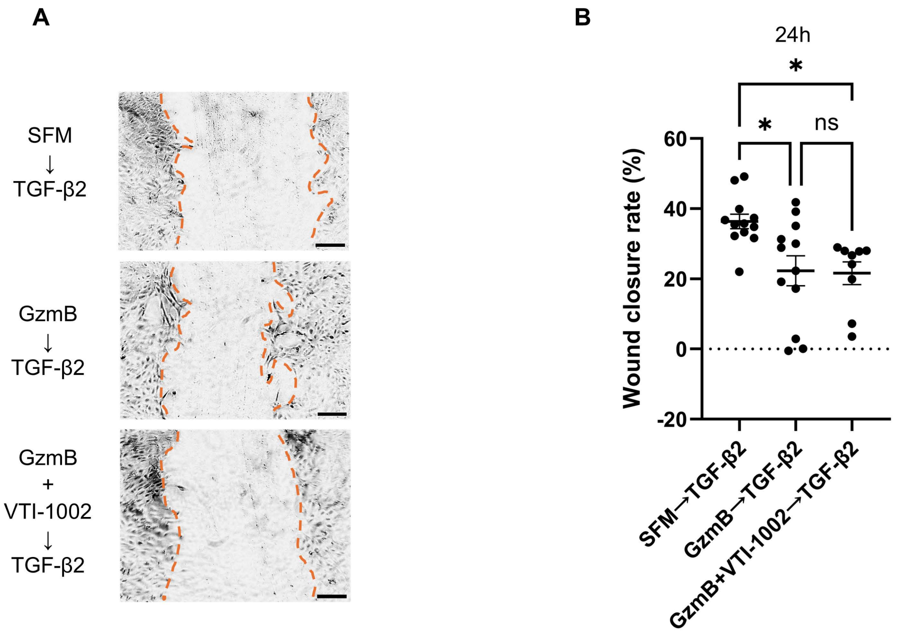

Supplemental Figure 7: Acute GzmB stimulation delays TGF-β2-mediated wound closure in ARPE-19 at 24 hours . A Representative phase-contrast images of scratch wounds in each treatment group at a 24-hour timepoint. The borders of initial wound edges are indicated by orange dotted lines. Scale bar, 500 μm. **B** ARPE-19 wound closure rate in each treatment group at a 24-hour timepoint (n = 9-12 samples per group; representative of three-independent experiments). The data are presented as mean ± SEM. Statistical analyses were performed by one-way ANOVA Tukey's multiple comparison test, $*p < 0.05$.

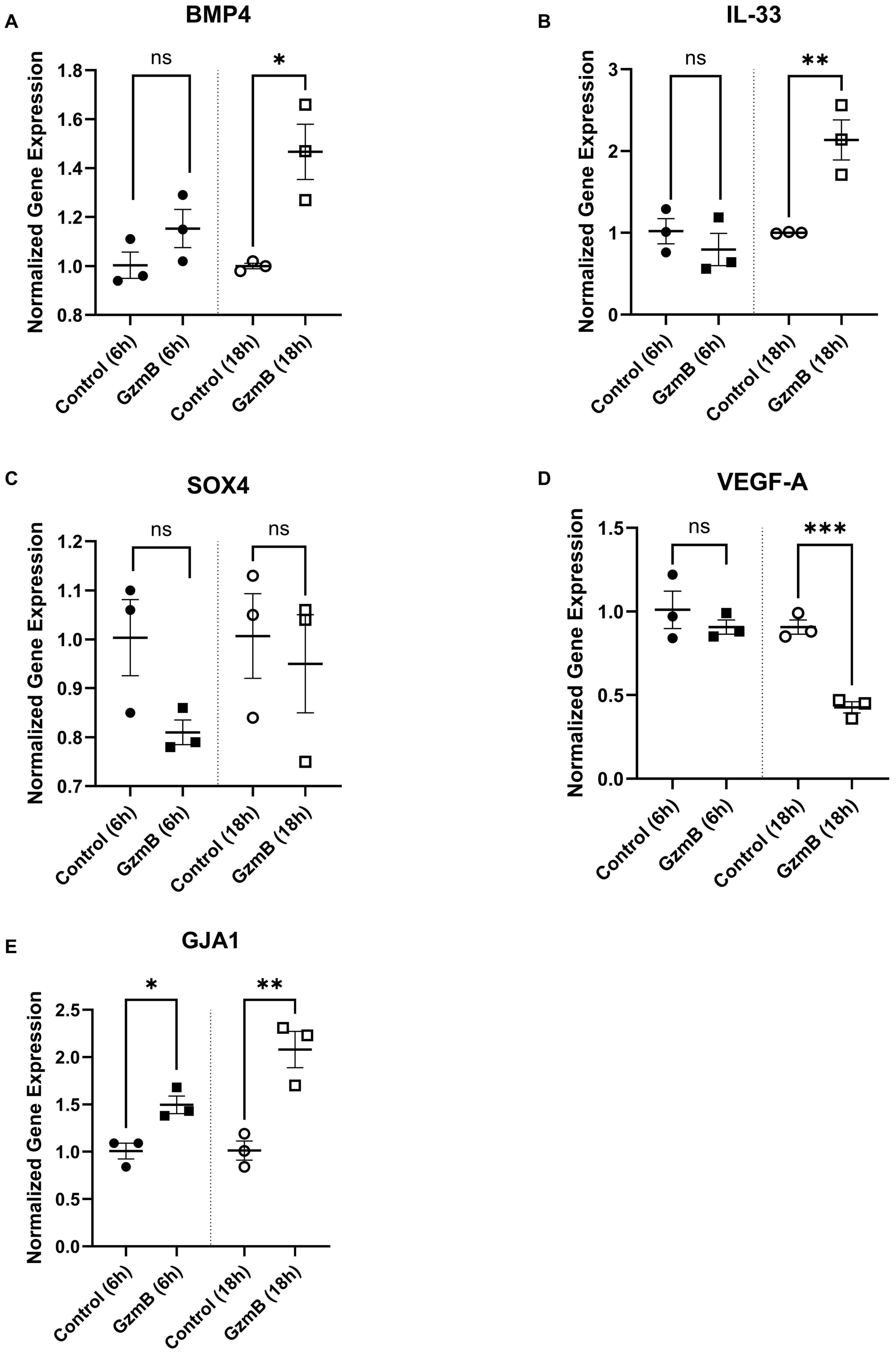

Supplemental Figure 8: GzmB modulates key genes involved in EMT, inflammation, angiogenesis and cell-cell interaction. A-E qPCR analysis of the gene expression of BMP4, IL-33, SOX4, VEGF-A and GJA1 in ARPE-19 at 6 or 18 hours after GzmB stimulation (n = 3 samples; representative of three independent experiments). The data are presented as mean ± SEM. Statistical analyses were performed by student's t-test, *$p < 0.05$, **$p < 0.01$, and ***$p < 0.001$.

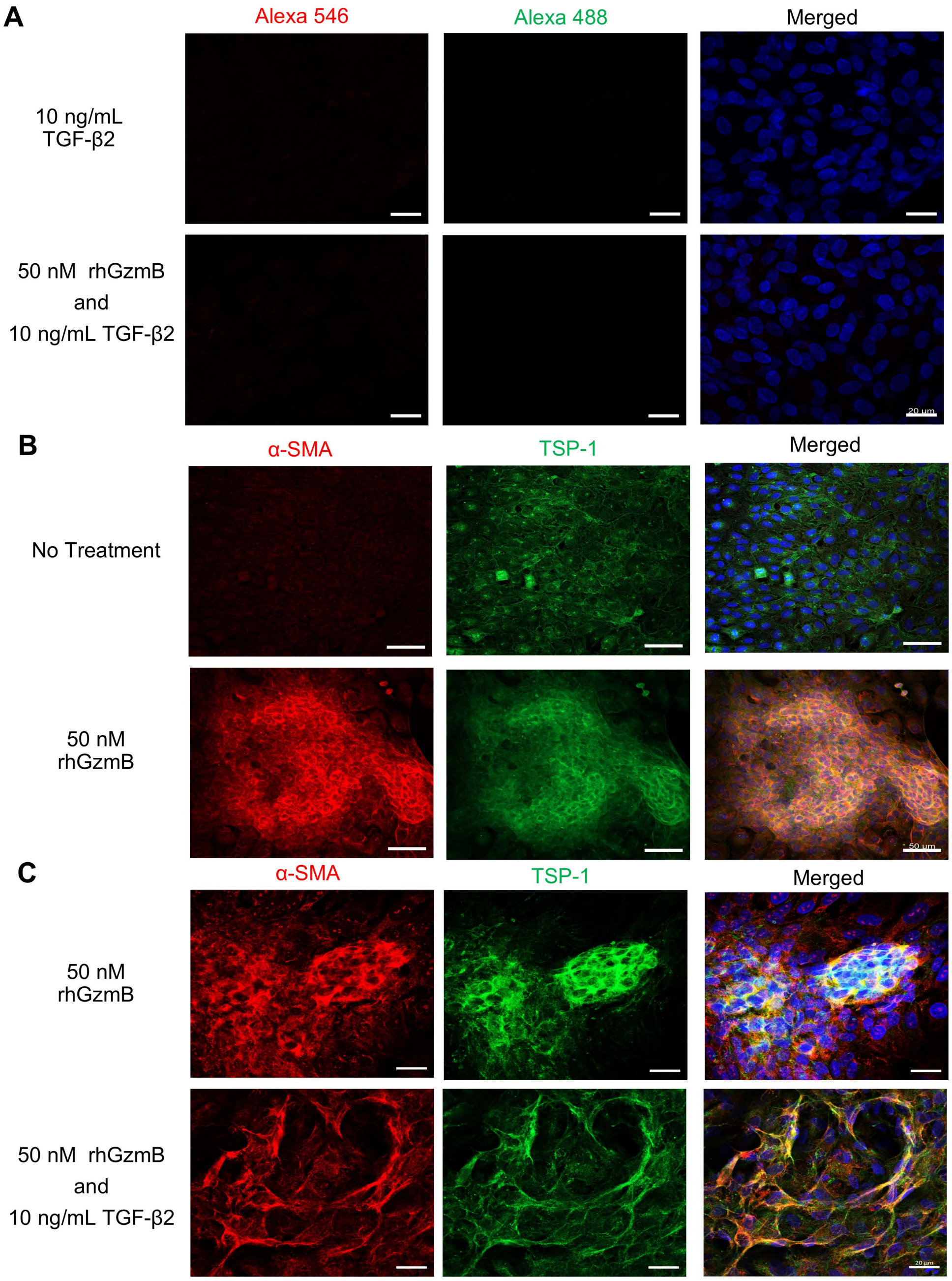

Supplemental Figure 9: GzmB promotes partial EMT in ARPE-19 cells and delays full transdifferentiation into myofibroblasts in the presence of TGF-β2. A Negative staining of ARPE-19 after 48-hour TGF-β2 treatment or after 6-hour 50 nM GzmB stimulation followed by 48-hour TGF-β2 treatment. **B** Immunolabeling of α-SMA (red), TSP-1 (green) and cell nuclei (blue) in ARPE-19 cells without any treatment or with 6-hour 50 nM GzmB stimulation. **C** Immunolabeling of α-SMA (red), TSP-1 (green) and cell nuclei (blue) with 50 nM GzmB stimulation or with 6-hour 50 nM GzmB stimulation followed by 48-hour TGF-β2 treatment. Scale bar, 20 μm.
